# MYC regulates the unfolded protein response and glucose and glutamine uptake in endocrine resistant breast cancer

**DOI:** 10.1186/1476-4598-13-239

**Published:** 2014-10-23

**Authors:** Ayesha N Shajahan-Haq, Katherine L Cook, Jessica L Schwartz-Roberts, Ahreej E Eltayeb, Diane M Demas, Anni M Warri, Caroline O B Facey, Leena A Hilakivi-Clarke, Robert Clarke

**Affiliations:** Lombardi Comprehensive Cancer Center and Department of Oncology, Georgetown University School of Medicine, 3970 Reservoir Road NW, Washington, DC, 20057 USA; University of Turku, Turku, Finland

**Keywords:** Breast cancer, Antiestrogen resistance, Metabolism, Glutamine, Glucose, MYC, Unfolded protein response

## Abstract

**Background:**

About 70% of all breast cancers are estrogen receptor alpha positive (ER+) and are treated with antiestrogens. However, 50% of ER + tumors develop resistance to these drugs (endocrine resistance). In endocrine resistant cells, an adaptive pathway called the unfolded protein response (UPR) is elevated that allows cells to tolerate stress more efficiently than in sensitive cells. While the precise mechanism remains unclear, the UPR can trigger both pro-survival and pro-death outcomes that depend on the nature and magnitude of the stress. In this study, we identified MYC, an oncoprotein that is upregulated in endocrine resistant breast cancer, as a regulator of the UPR in glucose-deprived conditions.

**Methods:**

ER+ human breast cancer cell lines (LCC1, LCC1, LY2 and LCC9) and rat mammary tumors were used to confirm upregulation of MYC in endocrine resistance. To evaluate functional relevance of proteins, siRNA-mediated inhibition or small molecule inhibitors were used. Cell density/number was evaluated with crystal violet assay; cell cycle and apoptosis were measured by flow cytometry. Relative quantification of glutamine metabolites were determined by mass spectrometry. Signaling molecules of the UPR, apoptosis or autophagy pathways were investigated by western blotting.

**Results:**

Increased MYC function in resistant cells correlated with increased dependency on glutamine and glucose for survival. Inhibition of MYC reduced cell growth and uptake of both glucose and glutamine in resistant cells. Interestingly, in glucose-deprived conditions, glutamine induced apoptosis and necrosis, arrested autophagy, and triggered the unfolded protein response (UPR) though GRP78-IRE1α with two possible outcomes: (i) inhibition of cell growth by JNK activation in most cells and, (ii) promotion of cell growth by spliced XBP1 in the minority of cells. These disparate effects are regulated, at different signaling junctions, by MYC more robustly in resistant cells.

**Conclusions:**

Endocrine resistant cells overexpress MYC and are better adapted to withstand periods of glucose deprivation and can use glutamine in the short term to maintain adequate metabolism to support cell survival. Our findings reveal a unique role for MYC in regulating cell fate through the UPR, and suggest that targeting glutamine metabolism may be a novel strategy in endocrine resistant breast cancer.

## Introduction

70% of all breast cancers are estrogen receptor α positive (ER+) and are treated with endocrine therapies (antiestrogens or aromatase inhibitors) that disrupt the ER function. The antiestrogens Tamoxifen (TAM) antagonizes estrogen binding to the ER while ICI 182,780 (ICI; Faslodex/Fulvestrant) targets ER for degradation. Despite their clear clinical activity, 50% of ER + tumors never respond or eventually develop resistance to antiestrogens [1, 2]. Understanding the molecular basis of endocrine resistance is a prerequisite to finding new interventions to resistance in the clinic.

c-MYC (hereafter referred to as MYC) is a transcription factor that is frequently deregulated in human cancers. MYC contributes to cancer progression through its involvement in several cellular functions including cell cycle progression, proliferation, differentiation, and apoptosis [3–6]. MYC is overexpressed in 30-50% of high-grade breast tumors [7, 8]. Activation of MYC is implicated in hormone-independence *in vitro* and endocrine resistance in patients [9], and it is predictive of a shorter time to recurrence following adjuvant TAM therapy [10]. The oncogenic activity of MYC depends on its ability to dimerize with MAX [11, 12]. Thus, agents that disrupt MYC-MAX heterodimers might be useful in treating some antiestrogen resistant breast cancers.

MYC controls several genes that regulate glycolysis and glutaminolysis [13, 14]. Both normal and cancer cells use glucose and glutamine to generate energy (ATP), produce raw materials for the synthesis of amino acids, fatty acids, and nucleosides, and maintain redox balance. However, rapidly growing cancer cells demand higher levels of substrates for macromolecule synthesis and for maintaining redox balance [15, 16]. Whether MYC can regulate cellular metabolism in antiestrogen resistant cancers, and whether this is a key component of this phenotype, remain unknown.

We describe how MYC upregulation in ER + antiestrogen resistant breast cancer cells increases dependency on glucose and glutamine but enables cell survival in glucose-deprived conditions by increasing dependency on glutamine. We show that glutamine in glucose-deprived conditions triggers the UPR through glucose-regulated protein-78 (GRP78/HSP5A/BiP) and inositol-requiring enzyme-1α (IRE1α/αRΝ1), and simultaneously, activates both pro-death and pro-survival pathways by increasing c-Jun N-terminal kinase (JNK) activation and spliced X-box protein-1 XBP1(s), respectively. While this UPR promotes apoptosis in most resistant cells in the short-term (72 h), in the longer term (>72 h), cell survival is promoted through cellular adaption to glutamine-only conditions in a minority of the cells that show adjusted MYC levels. Thus, safely targeting glutamine metabolism is a promising strategy to treat MYC-driven antiestrogen resistant breast cancer.

## Experimental procedures

### Cell culture and reagents

LCC1 (sensitive), LCC2 (TAM resistant; ICI sensitive), and LCC9 (ICI resistant and TAM cross-resistant) and LY2 (LY 117018 [Raloxifene analog] resistant and TAM and ICI cross-resistant) cells were established as previously described [17, 18]. Cells were grown in phenol red–free IMEM (Life Technologies, Grand Island, NY; A10488-01) with 5% charcoal-stripped calf serum (CCS); this media contains 2 mM L-glutamine and ~12 mM glucose. For glucose/glutamine-dependency assays, DMEM without glucose or glutamine (Life Technologies; A14430-01) was used supplemented with 5% CCS. LCC9Gln were derived from LCC9: cells were grown in DMEM without glucose but containing 2 mM L-glutamine (glutamine-only media) for 72 h; cells that survived (<5%) were continually grown in glutamine-only media for 12 weeks. All cells were authenticated by DNA fingerprinting and tested regularly for *Mycoplasma* infection. Faslodex and STF-31 were obtained from Tocris Bioscience (Ellisville, MO). Compound-968 was purchased from EMD Millipore (Billerica, MA). 10058-F4 was kindly provided by Dr. Steven Metallo (Georgetown University, Department of Chemistry). All other chemicals were purchased from Sigma-Aldrich.

### Western blot analysis

Total protein (~20 μg) was isolated from cells following 48 h treatment or vehicle control (0.02% DMSO or ethanol) for protein analysis as previously described [19]. The following antibodies were used: MYC, MAX, NBR1, p62/SQSTM1, GRP78, IRE1α, phospho-JNK(Thr183/Tyr185), JNK, CHOP, cleaved Caspase-7 (CASP7), LC3B, (Cell Signaling, Danvers, MA); p62/SQSTM1 (BD Biosciences, San Jose, CA); GLS (Abnova, Taipei City, Taiwan; Abcam, Cambridge, England); GLUL (Origene, Rockville, MD); BCL2 (Enzo Life Sciences, Farmingdale, NY), XBP1s (BioLegend, San Diego, CA) β-actin and β- tubulin (Santa Cruz Biotechnology, Santa Cruz, CA).

### Cell growth, apoptosis, necrosis, autophagy and reactive species assays

For determination of cell number, cells were plated in 96-well plates at 5 × 10^3^ cells/well. At 24 h, cells were treated with specified drugs for 48 h (or otherwise indicated). After treatment, media were removed, and plates were stained with a solution containing 0.5% crystal violet and 25% methanol, rinsed, dried overnight, and resuspended in citrate buffer (0.1 M sodium citrate in 50% ethanol). Intensity of staining, assessed at 570 nm and quantified using a VMax kinetic microplate reader (Molecular Devices Corp., Menlo Park, CA), is directly proportional to cell number [20]. For apoptosis and necrosis, cells were treated for 48 h, and stained with an Annexin V-fluorescein isothiocyanate and propidium iodide, respectively (Trevigen, Gaithersburg, MD). Autophagy was detected by detecting SQSTM1/p62 and LC3II proteins by Western blotting. For the reactive species assay, cellular levels of total reactive species (RS; kit measures both oxygen and nitrogen species) were determined using the Total ROS detection kit (Enzo Lifesciences) and measured by Flow Cytometry and Cell Sorting Shared Resources.

### Cell cycle analysis

Cells were cultured at 60-80% confluence in growth medium for 24 h. The following day, cells were treated with vehicle, ICI (100 nM), and/or 10058-F4 (25 μM) for an additional 72 h. Cells were then fixed in ethanol, and analyzed by the Flow Cytometry Shared Resource according to the method of Vindelov et al. [21].

### Transfection with siRNA or cDNA

Cells were plated at 60-80% confluence. 5 μM MYC siRNA (SMARTpool: ON-TARGETplus set of four MYC siRNA Dharmacon, Lafayette, CO), 10 GLS1, GRP78 (HSPA5), IRE1a or XBP1 (10 nM of 3 unique 27mer siRNA duplexes; Origene, Rockville, MD) or their respective control siRNA, were transfected using the TransIT-siQUEST (Mirus, Madison, WI) transfection reagent. At 48 h, 100 nM ICI or vehicle was added to the siRNA-transfected cells. For MYC overexpression, pcDNA3-MYC (plasmid 16011) was purchased from Addgene (Cambridge, MA) [22] and tranfected with TransIT-2020 (Mirus). Cells were lysed at 48 h post-transfection and subjected to Western blot analysis or cell number assay as described above.

### Transcription promoter-reporter assays

Cells were transfected with 0.4 μg of MYC luciferase reporter plasmid (plasmid 16601) from Addgene and 0.1 μg pCMV-Renilla (Promega, Madison, WI) per well using the TransIT-2020 transfection reagent. Activation of the luciferase constructs was measured at 48 h post-transfection using the Dual Luciferase Assay Kit (Promega). Luciferase values were normalized to Renilla luminescence. Three independent experiments were performed in quadruplicate. Data are presented as the mean ± SE for all experiments.

### Orthotopic xenografts in athymic mice

Five week old ovariectomized athymic nude mice (Harlan, Fredrick, MD) were injected orthotopically with 1.0 × 10^6^ LCC1/LCC9 cells in 50% Matrigel into mammary fat pads. 17β-estradiol supplementation from a subcutaneous, 0.72 mg pellet (Innovative Research of America) with 60-day release was used. Mice were sacrificed after 9 weeks, tumors were fixed in formalin, and processed using routine histological methods as previously described [23]. Mice were housed and maintained under specific pathogen-free conditions and used in accordance with institutional guidelines approved by Georgetown University Animal Care and Use Committee (GUACUC).

### Carcinogen-induced mammary tumors in rats

Mammary tumors were induced in 50-day-old female Sprague–Dawley (Harlan) rats with 7,12-dimethylbenz[a]anthracene (10 mg; DMBA; Sigma-Aldrich) by oral gavage. Tumor (15 ± 3 mm, long axis) bearing rats were switched to AIN-93G diet containing 337 ppm tamoxifen citrate (Harlan; 15 mg/kg/day TAM). Tumors were classified by growth responsiveness to TAM treatment. Sensitive tumors completely regressed or stopped growing with TAM treatment; Acquired Resistant tumors stopped or regressed but then re-grew after ≥4 weeks; and *de novo* Resistant tumors continued to grow during treatment. Animals were euthanized at 38 weeks. Tumors used in this study were confirmed as adenocarcinomas by histopathological evaluation (ARUP Laboratories, Utah, IL) [23]. Rats were housed and maintained under specific pathogen-free conditions and used in accordance with institutional guidelines approved by Georgetown University Animal Care and Use Committee (GUACUC).

### Immunohistochemistry (IHC)

Tumors were fixed in formalin for 24 h prior to embedding in paraffin. Immunostaining was performed on 5 μm thick sections with an antibody to MYC (1:500) or a non-specific negative control antibody using the diaminobenzidine (DAB) method and photographed using an Olympus BX61 DSU microscope at the Histopathology and Tissue Shared Resource.

### Relative metabolite quantification

Extracts from six biological replicates from LCC1 and LCC9 cells were spiked with internal standards and extracted using the method described by Sheikh *et al.* [24]. Samples were reconstituted in MeOH:H_2_O (1:1), and subsequently resolved on an Acquity ultra performance liquid chromatography (UPLC) column online with a triple quadrupole linear ion trap (QqQLIT) (Xevo-TQ-S, Waters Corporation, USA). The sample cone voltage and collision energies were optimized for each compound to obtain maximum ion intensity for parent and daughter ions using the “IntelliStart” feature of MassLynx software (Waters Corporation, USA). Data acquisition and analysis was done by the Proteomics and Metabolomics Shared Resource.

### Glutamine and glucose uptake

Glutamine and glucose uptake in LCC1 and LCC9 cells transfected with MYC siRNA was measured using a glutamine assay kit (BioAssay System, Hayward, CA); glucose uptake (2-NBDG, a fluorescently-labeled deoxy-glucose analog) was measured using a cell-based assay kit (#600470, Glucose uptake cell-based assay kit (Cayman Chemical, Ann Arbor, MI). In brief, differences in glucose or glutamine uptake, cells were transfected with MYC siRNA for 48 h. Glucose uptake was estimated by measuring the uptake of 2-NBDG by LCC1 and LCC9 cells in glucose-free media, as suggested by the protocol, for 30 min. Glutamine uptake was estimated by measuring the glutamine left in the media following the manufacturer’s protocol.

### Statistical analyses

Statistical analyses were performed using the Sigmastat software package (Jandel Scientific, SPSS, Chicago, IL). Where appropriate, relative cellular metabolites, protein expression, cell growth, and apoptosis were compared using either a Student’s *t* test or ANOVA with a *post hoc t*-test for multiple comparisons. Differences were considered significant at *p* ≤ 0.05. RI values were obtained by calculating the expected cell survival (*S*_exp_; the product of survival obtained with drug A alone and the survival obtained with drug B alone) and dividing *S*_exp_ by the observed cell survival in the presence of both drugs (*S*_obs_). *S*_exp_/*S*_obs_ > 1.0 indicates a synergistic interaction [25].

## Results

### MYC is upregulated in antiestrogen resistant breast cancer

**Figure 1:**
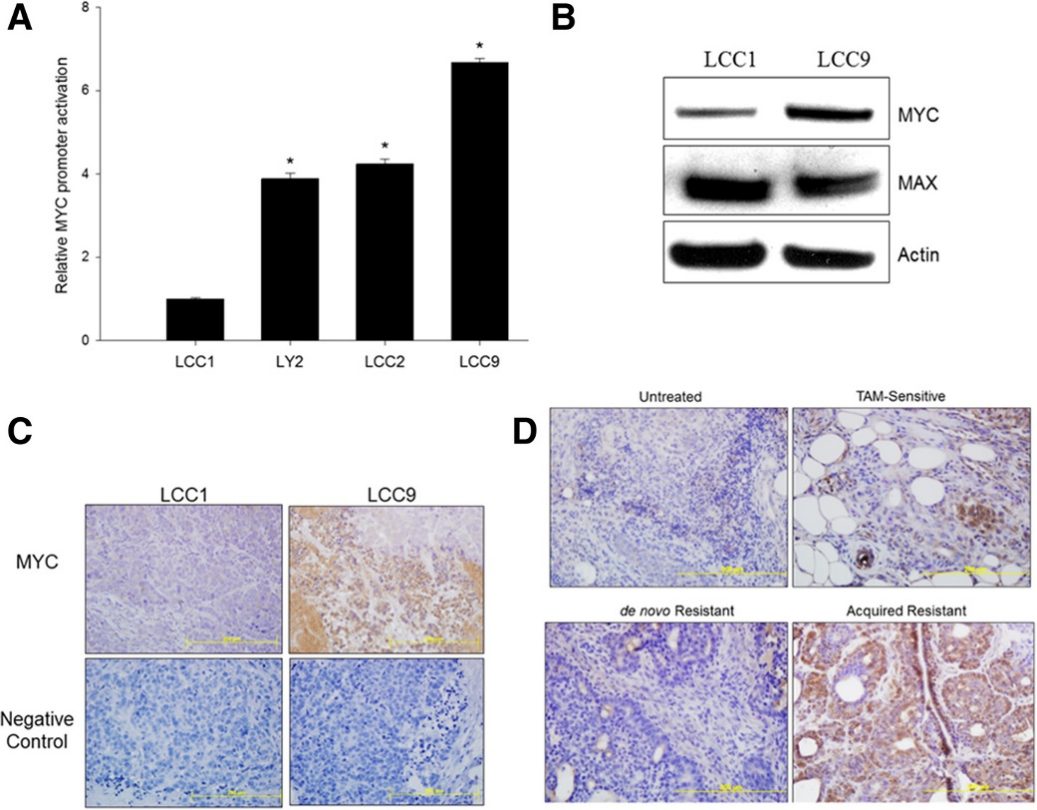
**MYC expression is elevated in antiestrogen resistant breast cancer** **in vitro** **and** **in vivo** **.** **A**, Basal MYC-luciferase activity is 4.24-fold (SE = 0.10) higher in LY2 and LCC2 (estrogen independent but responsive; antiestrogen resistant) and 6.67-fold (SE = 0.09) higher in LCC9 (estrogen independent and non-responsive; antiestrogen resistant) compared with LCC1 (estrogen independent but responsive; antiestrogen sensitive); see *Experimental Procedures*. ANOVA, *p* < 0.001; *p < 0.05 for MYC promoter activation in indicated cells compared with LCC1 cells. **B**, Western blot shows increased expression of MYC protein in LCC9 cells compared to LCC1 cells while MAX protein levels did not change; actin was used as a loading control. **C**, Immunohistochemical (IHC) MYC staining show increased protein levels (brown) in LCC9 compared with LCC1 xenografts; for negative controls, antibody diluents without MYC antibody were used. **D**, DMBA-induced rat mammary gland tumors with acquired resistance to TAM show increased levels MYC protein levels (brown) compared to sensitive (or *de novo* resistant) tumors.

MYC expression is increased in antiestrogen resistant breast tumors [10, 26]. To confirm activation of *MYC* gene in antiestrogen resistant cells, promoter luciferase activity was measured under basal conditions in ER + breast cancer cells that are either sensitive to antiestrogens (LCC1) or resistant to antiestrogens (LCC2, LY2 and LCC9). Relative to LCC1 cells, MYC promoter activation was 4-fold higher in LY2 and LCC2 cells and more than 6-fold higher in LCC9 cells (Figure 1A). Since the LCC9 cells showed the greatest upregulated MYC activation, LCC1 cells were compared with LCC9 cells for subsequent studies. Endogenous MYC protein was higher in LCC9 cells compared to LCC1 cells, while MAX levels remained unchanged (Figure 1B). In addition, untreated orthotopic xenografts showed upregulation of MYC protein in the antiestrogen resistant tumors (LCC9) (Figure 1C) when compared with sensitive tumors (LCC1). In the DMBA-induced rat mammary tumor model [23], MYC protein levels were higher in those tumors that acquired TAM resistance during treatment when compared with either TAM sensitive, *de novo* resistant, or untreated tumors (Figure 1D). These data strongly suggest that an increased MYC expression correlates with acquired antiestrogen resistance.

### Inhibition of MYC decreases cell growth in antiestrogen resistant cells

**Figure 2:**
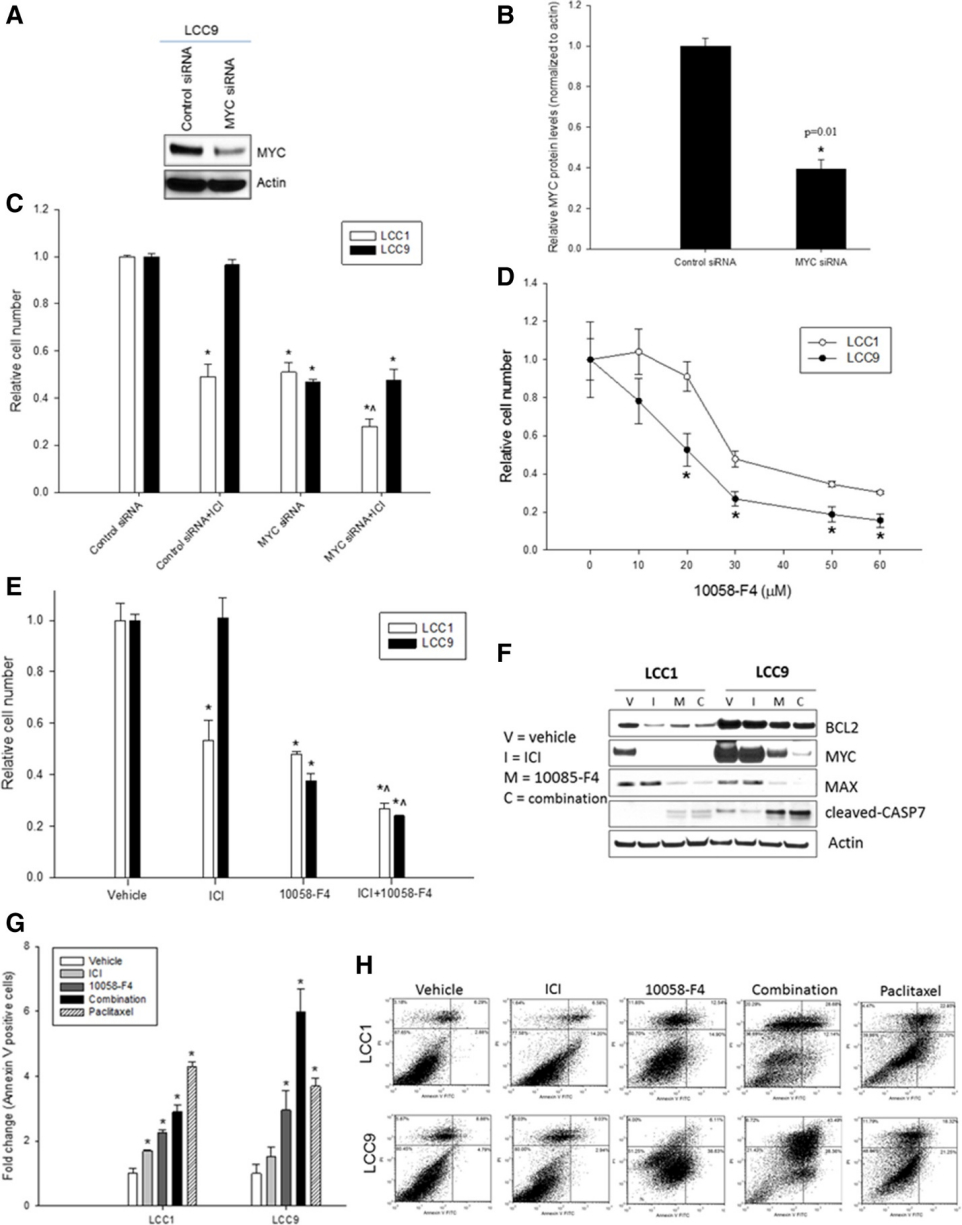
**MYC promotes survival in antiestrogen resistant cells.** **A**, Western blot, reduced MYC in LCC9 cells at 48 h with MYC siRNA compared to control siRNA. Actin is a loading control. **B**, Quantitation of MYC in in LCC9 cell show 60% reduction in MYC siRNA transfected cells compared with control siRNA. **C**, MYC siRNA interacted additively (RI = 1.11) with ICI in inhibiting cell number in LCC1 but not in LCC9 cells. Bars,mean ± SE of relative cell number (normalized to vehicle controls) for a representative experiment performed in sextuplicate. ANOVA, *p* < 0.001; *p < 0.05 for treatment versus control for respective cell lines. ^, *p* < 0.05 for LCC1 versus LCC9 cells with MYC siRNA + ICI. **D**, LCC9 cells showed increased sensitivity to 10058-F4compared with LCC1 cells at 48 h. Points, mean of cell number; bars, ±SE. **E**, 10058-F4 or ICI alone or the combination for 48 h inhibit cell number in LCC1. In LCC9 cells, RI = 1.51, suggest a modest synergistic interaction between ICI and 10058-F4; ANOVA, p < 0.001; *p < 0.05 for treatment versus control for respective cell lines. ^, *p* < 0.05 for ICI + 10058-F4 versus 10058-F4. **F**, Western blot show decrease in MYC, MAX, BCL2 and an increase in cleaved CASP7 , with 10058-F4 (MI: MYC inhibitor) or ICI + 10058-F4 (C: combination) compared with vehicle (V) alone or with ICI alone (I) treatment (48 h). **G**, Annexin V-FITC (apoptosis) in LCC1 and LCC9 cells with vehicle, ICI , 10058-F4 , or ICI + 10058-F4 (combination). ANOVA, p < 0.001; *p < 0.05 for indicated treatment versus vehicle control for respective cells lines. Paclitaxel, a positive control for apoptosis. **H**, Dot plots showcells positive for annexin-V-FITC (x-axis) and propidium iodide (PI; y-axis).

Knockdown of MYC with siRNA reduced MYC protein levels by 60% under basal conditions (results from LCC9 cells shown in Figure 2A and B) and significantly decreased cell number in both LCC1 and LCC9 cells compared with control siRNA (Figure 2C; *p* < 0.05). Treatment with ICI following MYC knockdown had an additive effect (RI = 1.11; see Experimental procedures) in LCC1 cells, while this combination did not further decrease cell number in LCC9 cells when compared with either treatment alone. LCC9 cells showed increased sensitivity to 10058-F4, a small molecule inhibitor of MYC-MAX heterodimer formation, compared with LCC1 cells at 48 h (Figure 2D). Cell number was significantly decreased for LCC9 cells treated with 20–60 μM of 10058-F4 compared with their LCC1 control cells (*p* < 0.05). In LCC1 cells, treatment with either 100 nM ICI or 25 μM 10058-F4 alone inhibited cell number; a combination of 10058-F4 and ICI significantly decreased cell number compared with the individual treatments (Figure 2E; *p* < 0.05). In LCC9 cells, while treatment with ICI had no effect, both 10058-F4 alone (*p* < 0.05) and a combination of ICI + 10058-F4 significantly (RI=1.51, a modest synergy) reduced the number of cells within 48 h (*p* < 0.05), suggesting a restoration of ICI sensitivity. Western blot analysis showed decreased levels of MYC, MAX, and BCL2 protein levels upon 10058-F4 treatments in both LCC1 and LCC9 cells (Figure 2F). LCC9 cells express lower levels of ERα under basal conditions compared with LCC1 cells [17, 27] and treatment with 10058-F4 alone did not change ERα levels. ICI, an antiestrogen that promotes degradation of ERα protein, and ICI + 10058 F4 decreased ERα levels (data not shown). Levels of cleaved Caspase-7 were highest in LCC9 cells treated with 10058-F4 and with the ICI + 10058-F4 combination, confirming induction of apoptosis under these conditions. 10058-F4 can decrease BCL2 protein levels [28]; BCL2 and other anti-apoptotic BCL2 proteins confer antiestrogen resistance in breast cancer cells [29]. Thus, the increased efficacy of 10058-F4, in comparison to MYC siRNA, in combination ICI may be due to a cumulative effect of its ability to downregulate MYC and other off-targets like BCL2.

### MYC inhibition induces apoptosis and cell cycle in resistant cells

**Figure 3:**
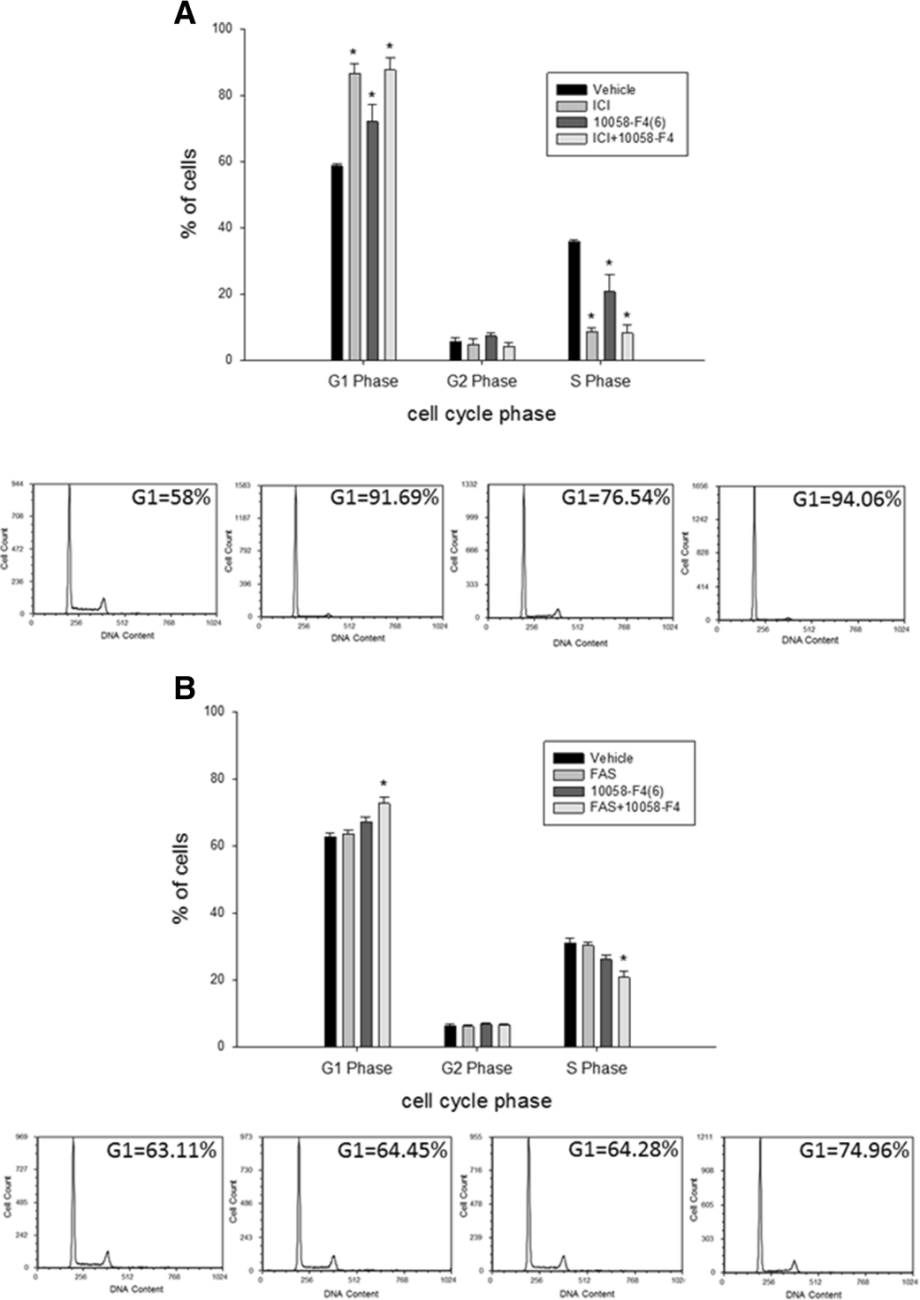
**Combination of MYC inhibitor and antiestrogen increased G1 cell cycle arrest in endocrine resistant cells.** **A**, *Top*, ICI (100 nM), 10058-F4 (25 μM), or the combination significantly increased percentage of cells in G1 arrest and reduced percentage of cells in S phase in LCC1 (*p* < 0.001). *Bottom*, Representative cell count plots for propidium iodide (PI) in LCC1 cells are shown. **B** *, Top*, Only the combination of ICI and 10058-F4 induced significant increase in G1 arrest in LCC9 cells (*p* < 0.001). *Bottom,* Representative cell count plots for PI in LCC9 cells are shown. Graphs represent data that are presented as the mean ± *SE* for three independent experiments. ANOVA, *p* < 0.001; *p < 0.001 for indicated treatment versus vehicle control.

To determine how 10058-F4 restored sensitivity of LCC9 cells to ICI, we studied changes in apoptosis. The proportion of cells undergoing apoptosis with combined ICI + 10058-F4 treatment was significantly higher in LCC9 compared with that in LCC1 cells (Figure 2G; *p* < 0.05). Dot plots for cells positive for apoptosis markers, Annexin-V-FITC and propidium iodide (PI), following different treatments are also shown in Figure 2H. Since MYC can regulate cell cycling [3], we analyzed the cell cycle profile of vehicle, 100 nM ICI, 25 μM 10058-F4, or the combination treatment at 48 h in LCC1 and LCC9 cells. ICI, 10058-F4, or the combination induced G1-phase cell cycle arrest in the antiestrogen sensitive LCC1 cells (Figure 3A). In the LCC9 cells (resistant), ICI or 10058-F4 treatment alone did not alter the cell cycle profile, whereas their combined treatment increased the percentage of cells in G1 arrest when compared with vehicle treated cells (Figure 3B; *p* < 0.001). These findings suggest that inhibition of MYC in LCC9 cells may restore sensitivity to ICI by both increasing apoptosis and inducing cell cycle arrest.

### MYC regulates glutamine and glucose uptake in antiestrogen resistant cells

**Figure 4:**
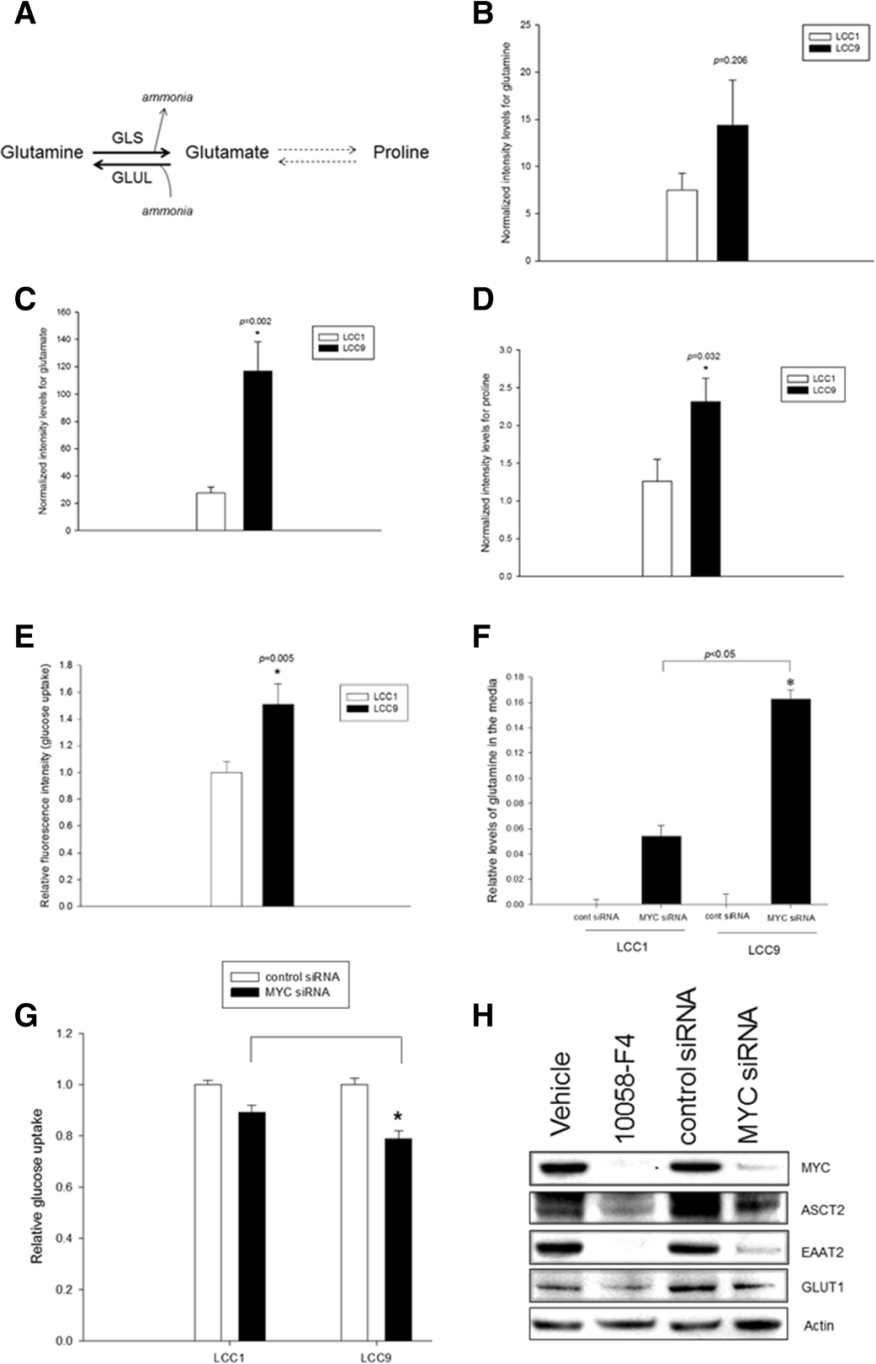
**Increased dependence on glutamine and glucose in antiestrogen resistant cells.** **A**, Schematic for glutamine metabolism: glutamine is converted to glutamate by the mitochondrial enzyme, glutaminase (GLS); the reverse reaction is catalyzed by glutamate-ammonia ligase (GLUL). Glutamine is an essential substrate for the biosynthesis of proline. **B-D**, Relative quantification of glutamine, glutamate, and proline by UPLC-QqQLIT showed a significant increase in glutamate (*p* = 0.002) and proline levels (*p* = 0.032) in LCC9 cells when compared with LCC1 control cells; six biological replicates from each cell line was used and levels of each respective metabolite was normalized to total protein levels in each sample. **E**, Uptake of glucose is significantly increased in LCC9 cells compared with LCC1 cells under basal conditions (*p* < 0.05). Relative cellular metabolites and glucose uptake were compared using Student’s *t* test **F-G**, Inhibition of MYC using siRNA significantly deceased (*F*) glutamine (*p* < 0.05) and (*G*) glucose uptake (*p* = 0.011) in LCC9 compared with LCC1 cells. ANOVA, *p* < 0.001. **H**, Inhibition of MYC with siRNA decreased protein levels of transporters of glutamine (ASCT2/SLC1A5), glutamate (EAAT2/SLC1A2) and glucose (GLUT1/SLC2A1) in LCC9 cells. Western blot shown is representative of three independent experiments.

Cancer cells with an aberrantly high expression of MYC often have deregulated cellular metabolism, particularly increased glycolysis and glutaminolysis [13]. To compare status of glutamine metabolism in LCC9 versus LCC1 cells, the relative concentration of glutamine metabolites were measured: glutamine, glutamate (immediate metabolite catalyzed by glutaminase, GLS and releasing ammonia; Figure 4A), and proline (downstream metabolite) using ultra performance liquid chromatography/mass spectrometry (UPLC/MS). While glutamine levels were not significantly different (*p* = 0.206), glutamate (*p* = 0.002), and proline levels (*p* = 0.032) were significantly higher in LCC9 compared with LCC1 cells (Figure 4B-D). In addition, uptake of glucose was significantly higher in LCC9 cells compared to LCC1 cells (Figure 4E; *p* = 0.005). Knockdown of MYC with siRNA inhibited cellular uptake of both glutamine (Figure 4F; *p* = 0.05) and glucose (Figure 4G; *p* = 0.011) more significantly in LCC9 cells than in LCC1 cells. Moreover, MYC knockdown reduced expression of glutamine transporter ASCT2 (SLC1A5), glutamate transporter EAAT2 (SLC1A2), and the glucose transporter GLUT1 (SLC2A1) in LCC9 cells (Figure 4H). Thus, MYC controls uptake of glutamine and glucose seen in antiestrogen resistant cells.

### Antiestrogen breast cancer cells show increased sensitivity to inhibitors of glutamine and glucose metabolism

**Figure 5:**
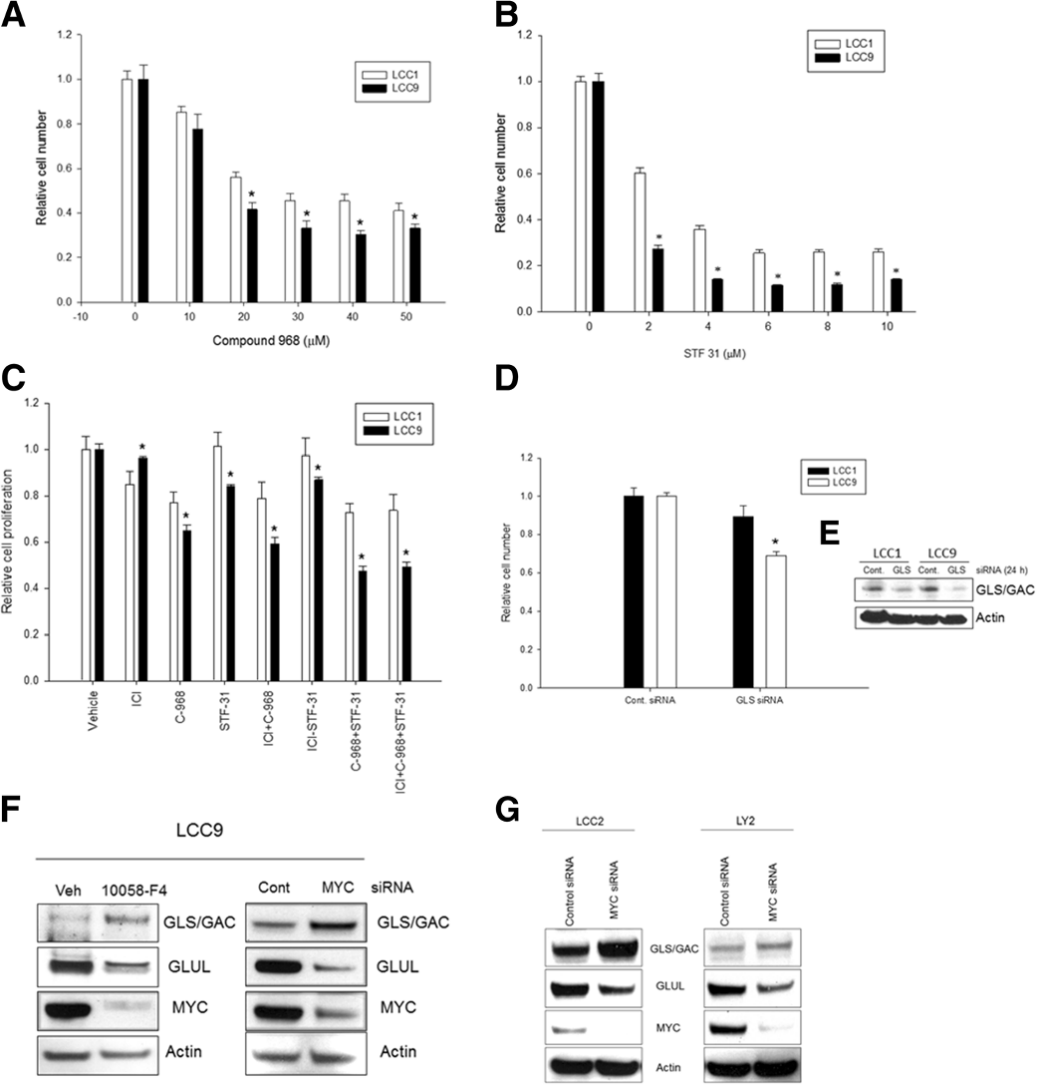
**Glutamine and glucose metabolism is increased in antiestrogen resistant cells.** **A-B**, LCC9 cells were significantly more sensitive to (*A*) compound-968, an inhibitor of GLS/GAC, and to (*B*) STF-31, an inhibitor of GLUT-1. Bars represent the mean ± SE of relative number (normalized to vehicle control) for a single representative experiment performed in sextuplicate. ANOVA, *p* ≤ 0.001; *p < 0.05 for LCC9 versus LCC1 for indicated concentrations. **C**, Cells were treated with compound-968 (20 μM), STF-31 (5 μM), ICI (100 nM), or the indicated combinations for 48 h. Bars represent the mean ± SE of relative cell number (normalized to vehicle controls) for a single representative experiment performed in sextuplicate. ANOVA, *p* < 0.001; *p < 0.05 for LCC9 versus LCC1 for indicated treatments. **D**, Knockdown of GLS levels with siRNA in LCC9 cells showed significant decrease in cell number within 24 h compared with that in LCC1 cells. ANOVA, *p* = 0.03; *p ≤ 0.05 for LCC9 GLS siRNA compared with LCC1 GLS siRNA. **E**, Western blot showing decreased levels of GLS in both cell lines; actin was used as a protein loading control. **F**, *Right*, LCC9 ells were treated with 10058-F4 (25 μM), or vehicle for 48 h; *left*, transfected with MYC or control siRNA for 48 h. Knockdown of MYC increased GLS/GAC levels and decreased GLUL levels. **G**, siRNA mediated MYC knockdown showed increase in GLS and a decrease in GLUL levels in LCC2 and LY2 cells.

Since LCC9 cells showed increased glutamine metabolism and glucose uptake, we determined whether inhibitors of these pathways differentially affected cell survival in LCC1 versus LCC9 cells. Cell number was significantly decreased in LCC9 compared with LCC1 cells in response to the GLS/GAC inhibitor compound-968 (Figure 5A; *p* < 0.05). Moreover, increasing doses of the GLUT1 inhibitor STF-31, an inhibitor of glycolysis, produced a significant decrease in cell number in LCC9 cells relative to LCC1 cells (Figure 5B; *p* < 0.05). While LCC9 cells showed significantly increased sensitivity to both STF-31 (*p* < 0.05) and compound-968 compared with LCC1 cells at 48 h (*p* < 0.05), adding ICI to either drug did not resensitize LCC9 cells to the antiestrogen (Figure 5C). Thus, specific inhibitors of glutamine and glucose metabolism are potent inhibitors of cell proliferation in both ER + sensitive and antiestrogen resistant breast cancer cells. Knockdown of GLS in LCC9 cells significantly decreased cell numbers within 24 h post transfection with GLS siRNA compared with that in LCC1 cells (Figure 5D). Western blot analysis of total GLS protein following siRNA mediated knockdown within 24 h is shown in Figure 5E.

GLS has two splice variants resulting from alternate splicing: KGA (~66 kDa; full-length) and GAC (~53 kDa; truncated form). GLS/GAC is the predominant form found in tumors [30] and is the variant present in the models used in this study. To show whether MYC regulates GLS/GAC levels in antiestrogen resistant cells, we inhibited MYC with siRNA or 10058-F4 in LCC9 (Figure 5E); and with MYC siRNA in LY2 and LCC2 cells (Figure 5F). In all three antiestrogen resistant cells, MYC inhibition increased GLS/GAC but inhibited glutamine synthase (GLUL), an enzyme that converts glutamate to glutamine. Thus, MYC can regulate GLS/GAC-GLUL enzyme levels to control glutamine metabolism in antiestrogen resistant cells.

### MYC increased sensitivity to deprivation of glutamine and glucose

**Figure 6:**
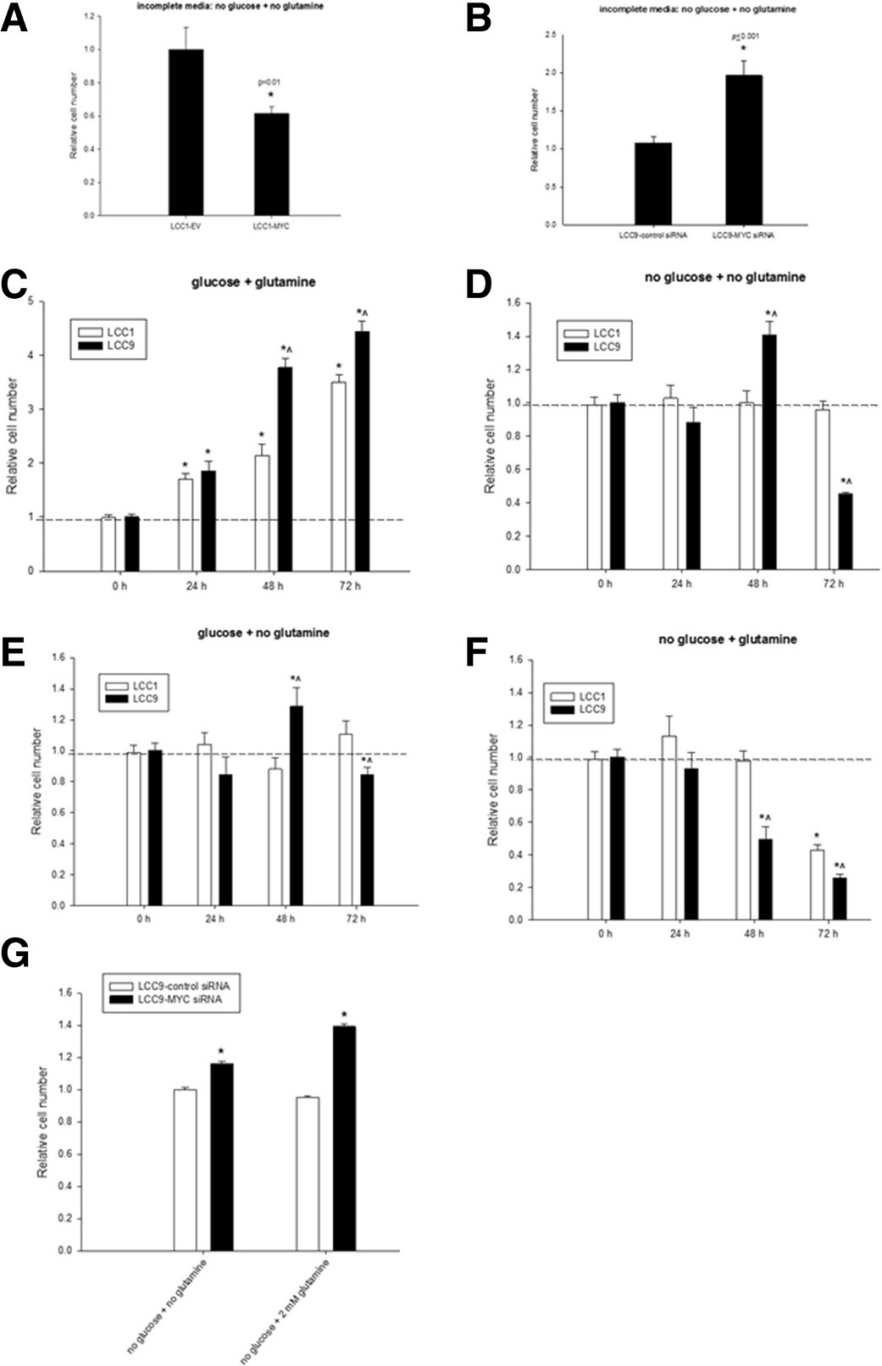
**MYC expression increases sensitivity to glucose and glutamine deprivation.** **A-B**, Overexpression of MYC in LCC1 cells significantly increased (*p* < 0.01) (A) and knockdown of MYC in LCC9 cells (B) significantly decreased cell number in the absence of glucose and glutamine (*p* ≤ 0.001). **(** **C-F** **)** LCC1 and LCC9 cells were grown in complete (12 mM glucose; 2 mM glutamine), incomplete (no glucose; no glutamine), glucose only (12 mM glucose; no glutamine), and glutamine-only (2 mM glutamine; no glucose) for 72 h. Changes in cell growth rates were determined by normalizing cell numbers measurements at 24 h, 48 h, and 72 h to cell numbers measurements at 0 h. At 72 h, LCC9 cells showed significantly higher growth rate compared to LCC1 in complete media. However, growth rate was significantly reduced for LCC9 in incomplete media when compared with LCC1 cells. In glucose-only media (at 72 h), LCC1 and LCC9 cells did not show an increase in cell growth. In glutamine-only media, LCC9 cells showed a significant decrease in cell number relative to LCC1 cells. *Dashed line* denotes change in scales between the graphs. Bars represent the mean ± SE of relative number (normalized to vehicle control) for a single representative experiment performed in sextuplicate. **G**, Knockdown of MYC in LCC9 cells reduced sensitivity to incomplete media, as seen in **B**, and also reduced inhibition of cell number in the presence of 2 mM glutamine in glucose-deprived conditions. ANOVA, *p* < 0.001; *p* ≤ 0.01 for LCC9-MYC siRNA versus LCC9-control siRNA for indicated treatment. Bars represent the mean ± SE of relative number (normalized to vehicle control) for a single representative experiment performed in sextuplicate.

To confirm whether MYC is responsible for the increased dependency on glutamine and glucose, MYC was either overexpressed in LCC1 cells (lower endogenous MYC expression/activation) or knocked down in LCC9 cells (higher endogenous MYC expression/activation) (*see* Figure 1). Figure 6A shows a significant decrease in cell number in LCC1 cells overexpressing MYC (*p* < 0.01), while Figure 6B shows a significant increase in cell survival is seen in LCC9 cells when MYC expression is reduced by RNAi (*p* ≤ 0.001) in the absence of both glucose and glutamine. Next, we determined number of LCC1 versus LCC9 cells in the presence or absence of glucose and glutamine at 24, 48, and 72 h. Cell growth was significantly greater in LCC9 compared with that in LCC1 cells at 48 and 72 h in complete media (Figure 6C; ANOVA *p* ≤ 0.001; *p* < 0.05). In incomplete media, LCC9 cells showed a significant increase in cell growth at 48 h compared with control (0 h; *p* < 0.05) or to LCC1 cells at 48 h (*p* < 0.05). However, at 72 h, cell growth in LCC9 was significantly decreased compared with control (p < 0.05) or LCC1 cells (Figure 6D; *p* < 0.05). In glucose-only conditions, LCC9 cells again showed an increase in cell growth at 48 h compared with either control (0 h) or LCC1 cells at 48 h. At 72 h, however, cell growth in LCC9 showed a significant decrease compared to either control (*p* < 0.05) or LCC1 cells at 72 h (Figure 6E; *p* < 0.05). Interestingly, in glutamine-only conditions, growth in LCC9 cells was significantly decreased compared with control or LCC1 cells at both 48 (*p* < 0.05) and 72 h (*p* < 0.05). LCC1 cells exhibited a similar but relatively slower response at 72 h when compared with the respective control (Figure 6F; *p* < 0.05). To delineate whether MYC directly regulated cell fate in the presence of glutamine-alone in glucose-deprived conditions, we investigated cell number following MYC inhibition in these conditions. Knockdown of MYC increased cell number in the absence of both glucose and glutamine in LCC9 cells as shown before in Figure 6B, and also when glutamine alone was present in glucose-deprived conditions, confirming the critical role of MYC (Figure 6G) in regulating cell fate in this condition.

### Glutamine-only conditions induces cell death and the UPR

**Figure 7:**
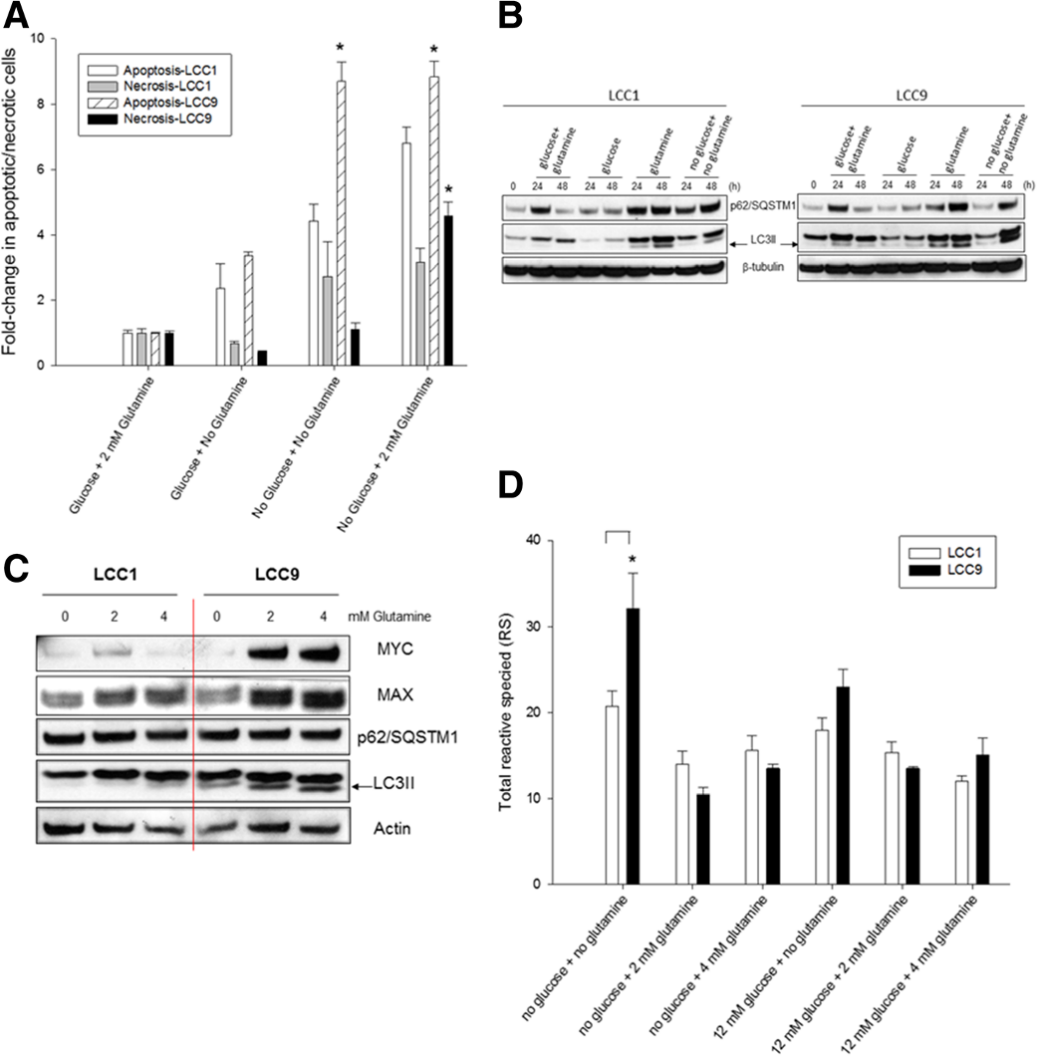
**Glutamine induces apoptosis and arrests autophagy via the UPR in glucose-deprived conditions.** **A**, Significantly higher levels of apoptosis were seen in LCC9 compared with LCC1 cells following treatment with 2 or 4 mM glutamine at 48 h. ANOVA, *p* < 0.05; *p < 0.05 for LCC9 versus LCC1 for indicated treatment. **B**, Time-course, 0, 24 and 48 h, analysis of the autophagosome-associated proteins LC3II (marker for autophagosome formation or enlargement) and p62/SQSTM1 (marker for autophagosome activity, degradation of cargo). Increased formation of autophagosomes but arrested cargo degradation was seen within 24 h in both LCC1 and LCC9 cells in glutamine only media (and in no-glucose + no glutamine) conditions at 24 and 48 h but not in glucose-only (or in glucose + glutamine) media. **C**, In presence of 2 or 4 mM glutamine at 48 h, LCC9 cells showed increased levels of MYC and MAX and LC3II but no change in SQSTM1/p62. **D**, Cellular levels of total reactive species (RS) was significantly elevated in LCC9 compared to LCC1 cells in incomplete media (ANOVA, *p* < 0.001; *p < 0.05 for LCC9 versus LCC1 with no glucose + no glutamine).

**Figure 8:**
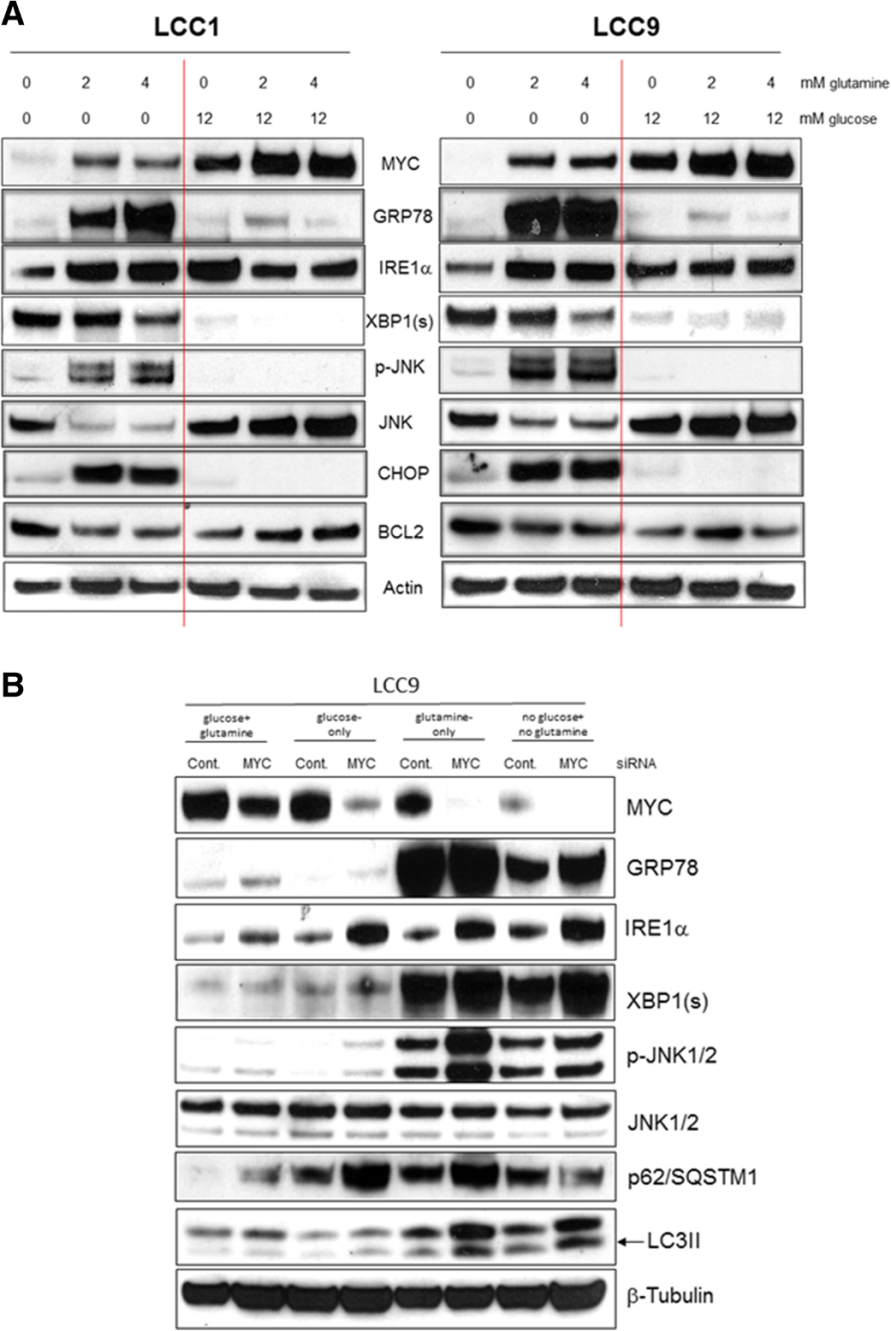
**Glutamine in glucose-deprived conditions activates the UPR.** **A**, Cells were plated at 70% confluence. 24 hr later, media was changed to 0, 2, or 4 mM glutamine alone or in presence of 12 mM glucose. *Western blot analysis showed* Increased levels of GRP78, IRE1a, phospho-JNK, CHOP and decreased levels of BCL2 were present in LCC1 (*right*) and LCC9 (*left*) cells in glutamine-only conditions. MYC protein levels were highest when both glucose and glutamine are present; MYC is undetectable when these metabolites are absent in the media. MYC expression in the presence of glutamine-only, but not in presence of glucose-only, conditions correlated with increased expression of UPR proteins. **B**, Knockdown of MYC for 24 h was followed by media change to either glucose + glutamine, glucose-only, glutamine-only or no glucose + no glutamine conditions for another 48 h. Western blots analysis showed that a decrease in MYC protein levels correlated with an increase in the UPR proteins IRE1α and phospho-JNK(Thr183/Ty4185), and the autophagosome formation marker LC3II, and the autophagosome cargo degradation marker p62/SQSTM1. GRP78 was also increased in glucose + glutamine, glucose-only and no glucose + no glutamine conditions but robust expression of GRP78 in glutamine-only conditions was not affected by MYC siRNA. Total levels of JNK did not change.

We next examined how the presence of glutamine in glucose-deprived conditions triggered a rapid decrease in cell number in antiestrogen resistant cells. To determine whether the decrease in cell survival in the presence of glutamine in glucose-deprived conditions was caused by induction of apoptosis, we measured apoptosis following 48 h of glutamine-only treatment in LCC1 and LCC9 cells. Apoptosis was significantly increased in LCC9 compared with LCC1 cells in the absence of both glutamine and glucose (Figure 7A). Moreover, in the presence of glutamine-only conditions, cells underwent significantly higher levels of apoptosis in LCC9 cells than in LCC1 cells. To determine autophagic flux, total protein from both LCC1 and LCC9 cells in the differ conditions (glucose + glutamine, glucose-only, glutamine-only, no glucose + no glutamine) were analyzed at 0, 24 and 48 h for p62/SQSTM1, LC3II and actin (Figure 7B). p62/SQSTM1 are adapter proteins that are autophagosome cargo markers used to determine activity within autolysosomes [31, 32]; however, each protein is selectively degraded by autophagy depending on the signaling cues and nature of stress [31]. An increase in LC3II expression is a marker of increased autophagosome formation and enlargement [33]. Increase in number of autophagosomes in the absence cargo degradation indicates interrupted autophagy that can promote apoptosis [34]. Moreover, Western blot analysis of total proteins from LCC9 cells treated with increasing concentrations of glutamine had higher levels of MYC, MAX and LC3II expression when compared with LCC1 cells; p62/SQSTM1 levels did not change (Figure 7C). Thus, while formation of autophagosomes may be triggered by the glutamine-only condition, autophagy-mediated degradation of cellular substrates is halted. Moreover, the induction of MYC suggests a possible role for this protein in regulating autophagy (see next section and Figure 8B). Disruption in cellular metabolic processes can lead to accumulation of reactive oxygen species (ROS) [35] and reactive nitrogen species (RNS) [36]. Figure 7D shows that deprivation of both glucose and glutamine significantly increased total reactive species (RS) levels in LCC9 cells. However, in both LCC1 and LCC9 cells, the presence of either glucose alone or glutamine alone did not change cellular RS levels compared with conditions where both metabolites are present. Thus, the decrease in cell number in glutamine-only conditions is independent of RS.

Induction of UPR has been reported in various cell models following a decrease in energy sources [37–39]. Western blot analyses of proteins associated with UPR showed increased GRP78, IRE1α, phospho-JNK(Thr183/Tyr185) and CHOP in glucose-deprived/glutamine-only conditions in LCC9 cells relative to LCC1 cells. Interestingly, while levels of MYC were highest when both glucose and glutamine are present, MYC is undetectable when these metabolites are absent. MYC expression in the presence of glutamine-only, but not in presence of glucose-only, conditions correlated with an increase in the UPR-related proteins. BCL2, an anti-apoptotic protein, was decreased in glucose-deprived glutamine only conditions (Figure 8A). No change in protein expression levels was detected for PERK or ATF6 (data not shown). GRP78, XBP1(s), and phospho-JNK were robustly induced in glutamine-only and no glucose + no glutamine conditions. Knockdown of MYC with siRNA (Figure 8B) increased: (i) GRP78 in all conditions (expect in glutamine-only conditions where high GRP78 expression likely prevented any effect of MYC siRNA on total GRP78 protein levels), (ii) IRE1α in all conditions, (iii) phospho-JNK (Thr183/Tyr185) in glutamine-only conditions without altering total JNK levels, and (iv) LC3II and p62/SQSTM1 levels in glutamine-only conditions. Thus, MYC directly controls the UPR and autophagy to control cell fate in ER + breast cancer cells under specific cellular signals that may be initiated by changes in intracellular glucose or glutamine.

### Induction of the UPR in glutamine-only conditions induces both pro-survival and pro-death signaling

**Figure 9:**
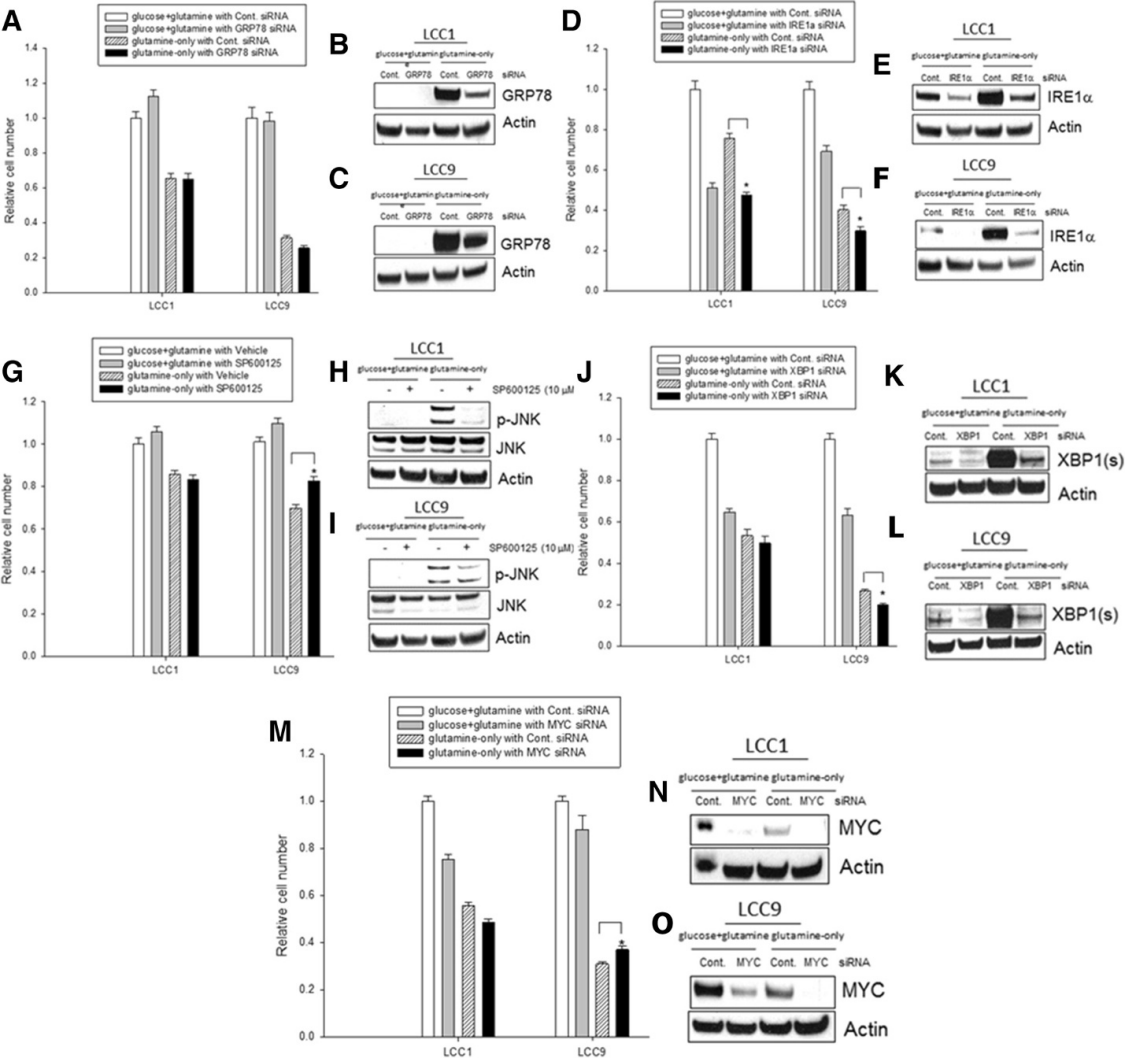
**UPR in glutamine-only conditions can lead to both pro-survival and pro-death outcomes.** Effect of transfection of siRNA targeting GRP78, IRE1α, XBP1(s), and MYC for 24 h; or JNK inhibition with a small molecule inhibitor (SP600125) on growth in either glucose + glutamine or glutamine-alone media. Western blot (48 h); **A-C**, GRP78. *D-F*, IRE1α. *G-I*, JNK. **J-L**, XBP1. *M-O*, MYC. Inhibition of GRP78 did not significantly further affect cell numbers in glutamine-only conditions in both LCC1 and LCC9 cell lines, *A*. Western blot analysis of total GRP78 protein are shown in both cell lines in different conditions, **B-C**. Knockdown of IRE1α, **D-F** and XBP1, **J-L**, significantly increased inhibition of cell growth in glutamine-only conditions in both cell lines. However, inhibition of JNK with SP600125 significantly decreased the inhibition of cell growth in glutamine-only conditions, **G-I**. Also, knockdown of MYC, **M-O**, significantly decreased inhibition of cell growth in glutamine-only conditions. Overall, MYC may have facilitate an IRE1α-XBP1 pathway to promote cell survival during glutamine-only conditions, and an IRE1α-phospho-JNK pathway to promote cell death in this condition. ANOVA, *p* ≤ 0.001; *p < 0.05 for respective cell lines transfected with indicated siRNA (or treated with SP600125, for JNK) compared with control siRNA (or vehicle alone, for JNK) in glutamine-only conditions.

Since the GRP78-IRE1α arm of the UPR is activated in glutamine-only conditions, we further investigated the role of these molecules in cell fate, especially since this particular pathway can drive both cell death via JNK activation, or cell survival via XBP1(s) splicing [37, 40, 41]. Knockdown of GRP78, IRE1α, XBP1, or MYC followed by growth in either glucose + glutamine or glutamine-alone media was compared (Figure 9A-F; J-O). SP600125, a small molecule inhibitor of JNK activation [42, 43] was used (Figure 9G-I) since we observed an increase in phospho-JNK (activation) in glutamine-only conditions (Figure 8A). Inhibition of GRP78 did not significantly affect the inhibition of cell number in glutamine-only conditions in both LCC1 and LCC9 cell lines (Figure 9A). Western blot analyses of total GRP78 protein are shown in both cell lines in different conditions in Figure 9B and C. Knockdown of IRE1α (Figure 9D; Westerns, E-F) and XBP1 (Figure 9J; Westerns, K-L) significantly increased inhibition of cell growth in glutamine-only conditions in LCC9 cells. XBP1 splicing to XBP1(s) by IRE1α promotes cell survival in breast cancer cells [23, 37, 41, 44–46], and thus, protein levels of XBP1(s) was determined. Inhibition of JNK activation with SP600125, however, significantly decreased the inhibition of cell growth in glutamine-only conditions (Figure 9J, Westerns, K-L). Finally, knockdown of MYC (Figure 9M, Westerns, N-O) significantly decreased inhibition of cell growth in glutamine-only conditions (as shown in Figure 6G). Thus, MYC may control an IRE1α-XBP1(s) pathway to promote survival during glutamine-only conditions, and also an IRE1α-phospho-JNK pathway to promote cell death under this condition; the balance between these two actions may determine individual cell fate.

### Prolonged exposure to glutamine-only conditions results in cell survival in a small number of endocrine resistant cells

**Figure 10:**
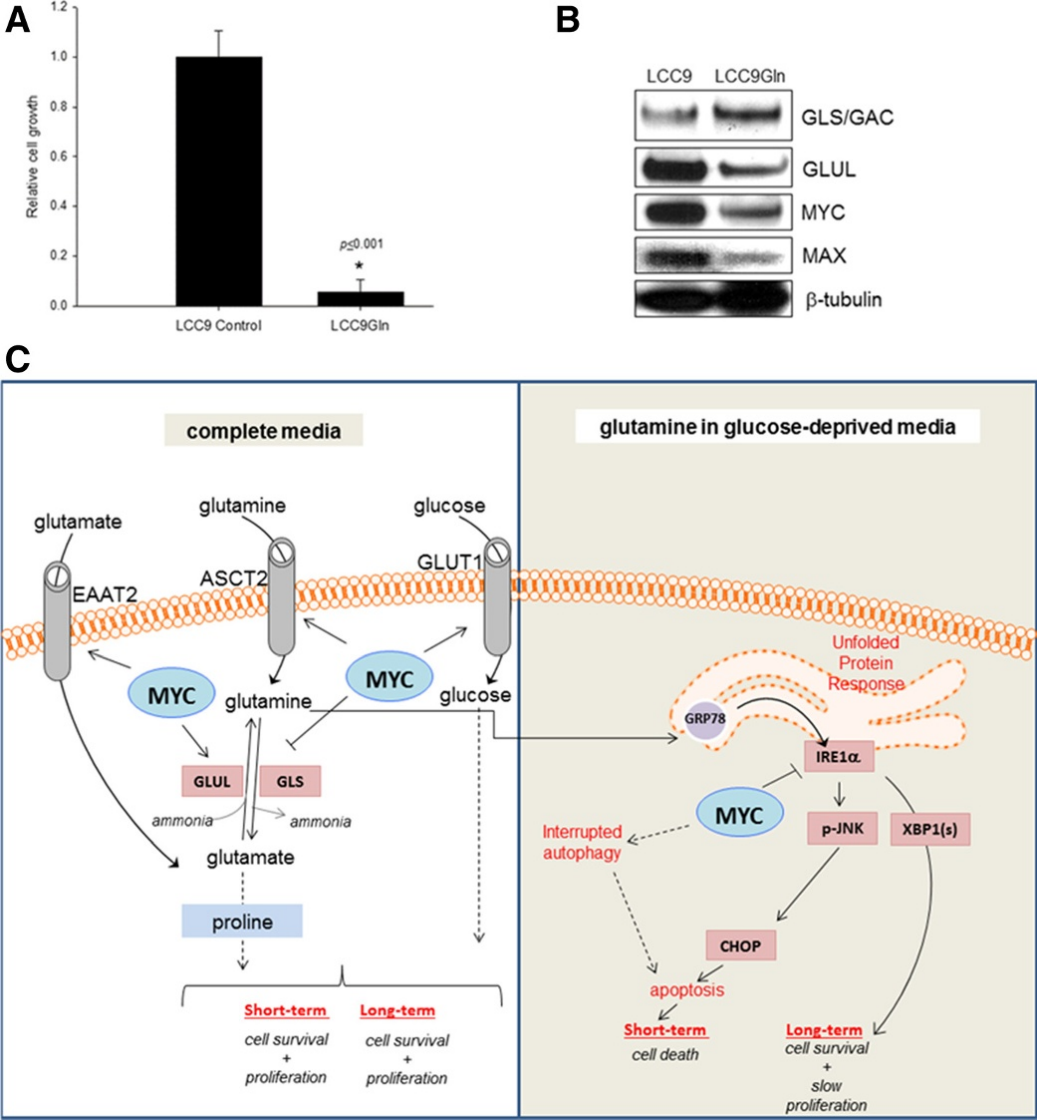
**MYC confers metabolic flexibility in antiestrogen resistant cells.** **A**, Rate of cell growth was significantly reduced in LCC9Gln cells compared with LCC9 control cells (*p* ≤ 0.001). Cell numbers at 72 h were compared using Student’s *t* test. **B**, MYC, MAX and GLUL protein levels were reduced, while GLS/GAC was increased, in LCC9Gln cells compared with control. **C**, Schematic diagram illustrating the role of MYC in regulating glutamine metabolism in complete (*right*; basal; with glucose and glutamine) and in glutamine-only conditions (*left*; glutamine but no glucose). MYC regulates glutamine, glutamate, and glucose uptake through transporters, ASCT2, EAAT2 and GLUT1, respectively, under normal conditions. In glucose-deprived conditions, glutamine metabolism triggers the UPR and induces cell death (inducing apoptosis and arresting autophagy) via a MYC-regulated IRE1α-JNK-CHOP in the short-term (72 h), and also promotes cell survival, through a IRE1α-XBP1(s); the surviving cells grow at a slower rate of cell proliferation (*A*), at >72 h. *Dashed line* denotes presence of intermediate metabolites/proteins that are not addressed in this study.

UPR is a complex adaptive mechanism that can have both pro-death and pro-survival outcomes in breast cancer cells [23, 37]. Since we detected both pro-survival XBP1(s) and pro-death (JNK) pathways in LCC9 cell in glutamine-only condition, we examined cell survival in these cells beyond 72 h. We followed cell growth in LCC9 cells beyond 72 h for all four conditions: (i) glutamine + glucose, (ii) no glucose + no glutamine, (iii) glucose + no glutamine, and (iv) no glucose + glutamine. While 100% of the cells survived in glutamine + glucose conditions, no cells survived in no glucose + no glutamine or glucose + no glutamine conditions. Most LCC9 cells underwent apoptosis in no glucose + glutamine conditions within 72 h, however, a small number (<5%) of cells survived. We followed the growth of these cells (LCC9Gln) for 12 weeks. Cell number in LCC9Gln cells was significantly slower than in LCC9 (control) cells grown in complete media (Figure 10A; *p* ≤ 0.001). Moreover, LCC9Gln cells showed an increased in GLS/GAC expression but a decrease in GLUL, MYC, and MAX expression (Figure 10B). Table 1 summarizes the levels of MYC protein and cell fate at 72 h (short-term) and >72 h (long-term) in LCC9 cells in the presence of glutamine and/or glucose. In summary, when glutamine and glucose are abundant, MYC promotes their uptake and uniquely controls GLS and GLUL expression in antiestrogen resistant breast cancer cells (Figure 10). In glucose-deprived conditions when glutamine is present, the UPR is triggered and apoptosis is induced through GRP78-IRE1α-JNK-CHOP within 72 h. However, a small number of cells use the UPR to maintain survival beyond 72 h through GRP78-IRE1α-XBP1(s), albeit at a lower growth rate, by adjusting MYC to promote glutamine metabolism.

**Table 1:** **Cell media conditions and corresponding levels of MYC and cell fate in antiestrogen resistant (LCC9) cells**

Cell media condition	MYC level; short-term cell fate (72 h)	MYC level; long-term cell fate (>72 h)
glucose + glutamine (basal)	High MYC; survival and proliferation; no UPR	High MYC; survival and proliferation; no UPR
no glucose + no glutamine	Low MYC; apoptosis, ROS/RNS; no UPR	No viable cells
glucose + no glutamine	High MYC; apoptosis; no UPR	No viable cells
no glucose + glutamine	High MYC; apoptosis; UPR	Low MYC; survival and slower rate of proliferation

## Discussion

MYC is a target of estrogen signaling in breast cancer cells [26] that can control diverse aspects of cancer cell survival including cellular metabolic reprogramming [47–49]. Activation of MYC has been linked to acquired antiestrogen resistance in human breast tumors [10] and poor clinical outcome [50]. Our findings show that MYC-driven pro-survival signaling in antiestrogen resistant breast cancer is partially dependent on proteins that control the cell cycle and apoptosis. While rapid drug metabolism limits the efficacy of 10058-F4 as an antitumor agent for solid tumors [28], its use *in vitro* showed that inhibiting MYC in antiestrogen resistant breast cancer cells confirmed the essential role of MYC activation in driving this phenotype. Metabolically stable small-molecule inhibitors of MYC hold significant promise as new agents to treat some drug resistant breast tumors.

MYC is an important regulator of glutamine and glucose metabolism [51]. Antiestrogen resistant breast cancer cells with higher MYC activation showed increased sensitivity to small molecule inhibitors of glutaminolysis and glycolysis (Figure 5C), but did not re-sensitize these cells to antiestrogens. Thus, activation of these metabolic pathways in resistant cells may be independent of ER-mediated signaling. Increased levels of glutamate and proline in antiestrogen resistant breast cancer cells imply an essential role for glutamine metabolism in sustaining cell survival. Glutamate, which is converted from glutamine by GLS, is an essential substrate for many cellular processes including for the formation of the antioxidant glutathione (GSH), feeding into the tricarboxylic acid (TCA) cycle via its metabolism to α-ketoglutarate (α-KG), indirect generation of NADPH for the synthesis of fatty acids and nucleotides, and a key source of the ammonia that is required for acid–base homeostasis [52, 53]. Conversely, a steady supply of glutamine is essential for cancer cells to modify proteins by O-linked N-acetylglucosamine (O-GlcNAc) through the hexosamine biosynthesis pathway. MYC can regulate global O-GlcNAc modification of proteins in rat fibroblast cells [54]. A fraction of glutamine is also used as the nitrogen donor for the *de novo* synthesis of purines and pyrimidines, needed to match the demands of nucleic acid production during cell proliferation, the rate of which is often greater in drug resistant cancer cells [52, 55]. Regulation of the GLS/GAC-GLUL system by MYC in antiestrogen resistant cells may, therefore, be essential to maintain and/or drive the resistant phenotype. MYC regulation of GLS and GLUL in antiestrogen resistant breast cancer cells was unexpected. While in prostate cancer cells, MYC knockdown was shown to decrease GLS and increase GLUL protein levels [56], in our antiestrogen resistant breast cancer cell models (LCC9, LCC2, and LY2) we observed the reverse effect – MYC knockdown increased GLS and decreased GLUL protein levels (Figure 5E and F).

The UPR pathway is an evolutionarily conserved adaptive pathway coupled to endoplasmic reticulum stress that is upregulated in antiestrogen resistant breast cancer [37]. Previously, we have shown that GRP78, a member of the HSP70 family of proteins, is overexpressed in antiestrogen resistant breast cancer cells and tumors and promotes their survival [23]. To date, it is unclear how the UPR regulates cellular metabolism or vice versa. Our findings show that GRP78, IRE1α, phospho-JNK and XBP1(s) are robustly upregulated in antiestrogen resistant ER + breast cancer cells in the presence of glutamine but absence of glucose (Figure 8A). While blocking JNK activation significantly reduced inhibition of cell growth in glutamine-only conditions, knockdown of XBP1 significantly increased the inhibition of cell growth (Figure 9). MYC directly inhibited phospho-JNK in glutamine-only conditions (Figure 8B). JNK or stress activated protein kinases (SAPK) belong to the MAPK family of proteins [57] and can directly contribute to pro-apoptotic signaling by phosphorylating and inactivating BCL2. In contrast, MYC inhibited IRE1α expression similarly in all four conditions of glucose and glutamine availability. Thus, regulation of JNK by MYC may reflect a mechanism to regulate the UPR under specific cellular stresses.

JNK can regulate MYC through phosphorylation [58] and can associate with and mediate MYC ubiquitination and degradation [59]. Moreover, in HeLa and HEK293 cells, MYC knockdown decreased LC3II levels and decreased formation of autophagosomes by inhibiting JNK [60]. In our endocrine resistant breast cancer cell models, MYC inhibition increased both JNK activation and LC3II levels, with an associated increased inhibition of cell growth in glutamine-only conditions (Figure 8B; Figure 9M). Further studies are needed to investigate how MYC controls stress signaling mediated through JNK and cell death pathways. Autophagosome formation and the accumulation of p62/SQSTM1 (Figure 7B) can trigger cell death through apoptosis during cellular stress [34], likely reflecting the inability to use autophagosome content degradation to feed intermediate metabolism. Thus, cellular metabolic status are clearly important in triggering specific MYC-mediated functions. Within a tumor, cancer cells can experience glucose deprivation due to an inadequate vasculature [61] or drug treatment [62]. Short-term inhibition of glycolysis may initiate UPR-mediated responses that subsequently induce apoptosis in most cells but can also promote survival in a small fraction of cells until an adequate energy supply becomes available to enable both cell survival and proliferation. Indeed, in bortezomib-induced cell death, MYC has been shown to bind to pro-apoptotic BCL2 proteins, NOXA and BIM, and cooperate with EGR1 [63]. Thus, MYC induced cell death in cancer cells warrants further elucidation.

Increased activation of MYC in antiestrogen resistant cells is also associated with their increased dependence on glutamine and glucose for cell survival. However, the presence of glutamine in glucose deprived conditions initiated an UPR-mediated pathway that killed most cells via apoptosis but allowed the survival of a small minority. In LCC9Gln cells, which survived in media containing glutamine but no glucose, MYC levels were reduced and GLS/GAC levels were increased when compared with the parental antiestrogen resistant LCC9 cells. These adaptations may ensure the appropriate balance between the levels of glutamine versus glutamate needed for the cells to survive in glucose-deprived conditions. Glutamine alone can sustain survival of a small cell population in the absence of glucose, albeit with a significantly decreased rate of cell proliferation (Figure 10A). Molecular characterization of the multiple passages of LCC9Gln versus parental cells is underway and will help elucidate the MYC-mediated and UPR-regulated adaptive pathway.

Excessive systemic energy demand in cancer can lead to cachexia, which affects a large number of cancer patients and results in the progressive loss of muscle and adipose tissue mass [64]. To date, it is unclear how therapeutic interventions can safely alter the energy demand of cancer cells within tumors without necessarily inducing additional metabolic problems for the host. While a tumor-to-liver Cori cycle is implicated in meeting glucose demands, a tumor-to-muscle cycle is implicated in meeting the glutamine demands of growing tumors [52, 64, 65]. In addition, fibroblasts in the tumor stroma can also supply tumor cells with glutamine [66]. As cancer progresses to a more aggressive, metastatic, drug resistant phenotype, the potential to induce cachexia likely also increases. Understanding the adaptation of cellular metabolism associated with drug resistant disease may offer new interventions to address this co-morbidity evident in many advanced cancers.

MYC expression is deregulated in various cancer types. Our findings show that antiestrogen resistant breast cancer cells express higher levels of MYC protein compared with sensitive cells, and elevated MYC levels correlate with increased sensitivity to deprivation of glutamine and glucose. While the levels of glutamine metabolites are higher in resistant cells, MYC regulates GLS/GAC and GLUL to meet the demands of the resistant phenotype, particularly during periods of glucose deprivation/insufficiency. Thus, glutamine metabolism may allow cancer cells to adapt to changes in glucose availability by re-programming existing pathways through MYC and the UPR. Safely targeting the glucose or glutamine pathway and/or the UPR could offer novel strategies to treat antiestrogen resistant breast cancer.

## Conclusions

MYC activation in endocrine resistant breast cancer cells increased their dependency on glutamine and glucose. However, when challenged with glucose deprivation, the presence of glutamine augmented MYC regulated the UPR with both: (i) a pro-death signaling through GRP78-IRE1α-JNK, that induced cell death in most cells, and (ii) a pro-survival signaling through GRP78-IRE1α-XBP1, that allowed a subset of cells to adapt and survive. Thus, targeting these pro-survival pathways may prevent the progression of some endocrine dependent cells to an endocrine resistant phenotype.

## Abbreviations

JNK: c-Jun N-terminal kinase
DMBA: 7,12-dimethylbenz[a]anthracene
ER+: Estrogen receptor α positive
ICI: ICI 182,780/Faslodex
GLS: Glutaminase
GLUL: Glutamine synthase
GRP78: Glucose-regulated protein 78
IRE1α: Inositol-requiring protein 1 α
RS: Reactive species
TAM: Tamoxifen
UPR: Unfolded protein response
XBP1(s): X-box-protein-1 spliced.

## References

[1] Clarke R, Skaar T, Leonessa F, Brankin B, James M, Brunner N, Lippman ME (1996). Acquisition of an antiestrogen-resistant phenotype in breast cancer: role of cellular and molecular mechanisms. Cancer Treat Res.

[2] Clarke R, Liu MC, Bouker KB, Gu Z, Lee RY, Zhu Y, Skaar TC, Gomez B, O’Brien K, Wang Y, Hilakivi-Clarke LA (2003). Antiestrogen resistance in breast cancer and the role of estrogen receptor signaling. Oncogene.

[3] Amati B, Alevizopoulos K, Vlach J (1998). Myc and the cell cycle. Front Biosci.

[4] Chen Y, Olopade OI (2008). MYC in breast tumor progression. Expert Rev Anticancer Ther.

[5] Dang CV (2012). MYC on the path to cancer. Cell.

[6] Planas-Silva MD, Bruggeman RD, Grenko RT, Smith JS (2007). Overexpression of c-Myc and Bcl-2 during progression and distant metastasis of hormone-treated breast cancer. Exp Mol Pathol.

[7] Blancato J, Singh B, Liu A, Liao DJ, Dickson RB (2004). Correlation of amplification and overexpression of the c-myc oncogene in high-grade breast cancer: FISH, in situ hybridisation and immunohistochemical analyses. Br J Cancer.

[8] Deming SL, Nass SJ, Dickson RB, Trock BJ (2000). C-myc amplification in breast cancer: a meta-analysis of its occurrence and prognostic relevance. Br J Cancer.

[9] McNeil CM, Sergio CM, Anderson LR, Inman CK, Eggleton SA, Murphy NC, Millar EK, Crea P, Kench JG, Alles MC, Gardiner-Garden M, Ormandy CJ, Butt AJ, Henshall SM, Musgrove EA, Sutherland RL (2006). c-Myc overexpression and endocrine resistance in breast cancer. J Steroid Biochem Mol Biol.

[10] Miller TW, Balko JM, Ghazoui Z, Dunbier A, Anderson H, Dowsett M, Gonzalez-Angulo AM, Mills GB, Miller WR, Wu H, Shyr Y, Arteaga CL (2011). A gene expression signature from human breast cancer cells with acquired hormone independence identifies MYC as a mediator of antiestrogen resistance. Clin Cancer Res.

[11] Dang CV, Lewis BC (1997). Role of oncogenic transcription factor c-Myc in cell cycle regulation, apoptosis and metabolism. J Biomed Sci.

[12] Nair SK, Burley SK (2003). X-ray structures of Myc-Max and Mad-Max recognizing DNA. Molecular bases of regulation by proto-oncogenic transcription factors. Cell.

[13] Dang CV (2011). Therapeutic targeting of Myc-reprogrammed cancer cell metabolism. Cold Spring Harb Symp Quant Biol.

[14] Gao P, Tchernyshyov I, Chang TC, Lee YS, Kita K, Ochi T, Zeller KI, De Marzo AM, Van Eyk JE, Mendell JT, Dang CV (2009). c-Myc suppression of miR-23a/b enhances mitochondrial glutaminase expression and glutamine metabolism. Nature.

[15] Teicher BA, Linehan WM, Helman LJ (2012). Targeting cancer metabolism. Clin Cancer Res.

[16] Ward PS, Thompson CB (2012). Metabolic reprogramming: a cancer hallmark even warburg did not anticipate. Cancer Cell.

[17] Brunner N, Boulay V, Fojo A, Freter CE, Lippman ME, Clarke R (1993). Acquisition of hormone-independent growth in MCF-7 cells is accompanied by increased expression of estrogen-regulated genes but without detectable DNA amplifications. Cancer Res.

[18] Brunner N, Boysen B, Jirus S, Skaar TC, Holst-Hansen C, Lippman J, Frandsen T, Spang-Thomsen M, Fuqua SA, Clarke R (1997). MCF7/LCC9: an antiestrogen-resistant MCF-7 variant in which acquired resistance to the steroidal antiestrogen ICI 182,780 confers an early cross-resistance to the nonsteroidal antiestrogen tamoxifen. Cancer Res.

[19] Shajahan AN, Wang A, Decker M, Minshall RD, Liu MC, Clarke R (2007). Caveolin-1 tyrosine phosphorylation enhances paclitaxel-mediated cytotoxicity. J Biol Chem.

[20] Shajahan AN, Dobbin ZC, Hickman FE, Dakshanamurthy S, Clarke R (2012). Tyrosine-phosphorylated caveolin-1 (Tyr-14) increases sensitivity to paclitaxel by inhibiting BCL2 and BCLxL proteins via c-Jun N-terminal kinase (JNK). J Biol Chem.

[21] Vindelov LL, Christensen IJ, Nissen NI (1983). A detergent-trypsin method for the preparation of nuclei for flow cytometric DNA analysis. Cytometry.

[22] Ricci MS, Jin Z, Dews M, Yu D, Thomas-Tikhonenko A, Dicker DT, El-Deiry WS (2004). Direct repression of FLIP expression by c-myc is a major determinant of TRAIL sensitivity. Mol Cell Biol.

[23] Cook KL, Shajahan AN, Warri A, Jin L, Hilakivi-Clarke LA, Clarke R (2012). Glucose-regulated protein 78 controls cross-talk between apoptosis and autophagy to determine antiestrogen responsiveness. Cancer Res.

[24] Sheikh KD, Khanna S, Byers SW, Fornace A, Cheema AK (2011). Small molecule metabolite extraction strategy for improving LC/MS detection of cancer cell metabolome. J Biomol Tech.

[25] Romanelli S, Perego P, Pratesi G, Carenini N, Tortoreto M, Zunino F (1998). In vitro and in vivo interaction between cisplatin and topotecan in ovarian carcinoma systems. Cancer Chemother Pharmacol.

[26] Musgrove EA, Sergio CM, Loi S, Inman CK, Anderson LR, Alles MC, Pinese M, Caldon CE, Schutte J, Gardiner-Garden M, Ormandy CJ, McArthur G, Butt AJ, Sutherland RL (2008). Identification of functional networks of estrogen- and c-Myc-responsive genes and their relationship to response to tamoxifen therapy in breast cancer. PLoS One.

[27] Cook KL, Clarke PA, Parmar J, Hu R, Schwartz-Roberts JL, Abu-Asab M, Wärri A, Baumann WT, Clarke R (2014). Knockdown of estrogen receptor-alpha induces autophagy and inhibits antiestrogen-mediated unfolded protein response activation, promoting ROS-induced breast cancer cell death. FASEB J.

[28] Guo J, Parise RA, Joseph E, Egorin MJ, Lazo JS, Prochownik EV, Eiseman JL (2009). Efficacy, pharmacokinetics, tisssue distribution, and metabolism of the Myc-Max disruptor, 10058–F4 [Z, E]-5-[4-ethylbenzylidine]-2-thioxothiazolidin-4-one, in mice. Cancer Chemother Pharmacol.

[29] Crawford AC, Riggins RB, Shajahan AN, Zwart A, Clarke R (2010). Co-inhibition of BCL-W and BCL2 restores antiestrogen sensitivity through BECN1 and promotes an autophagy-associated necrosis. PLoS One.

[30] Elgadi KM, Meguid RA, Qian M, Souba WW, Abcouwer SF (1999). Cloning and analysis of unique human glutaminase isoforms generated by tissue-specific alternative splicing. Physiol Genomics.

[31] Johansen T, Lamark T (2011). Selective autophagy mediated by autophagic adapter proteins. Autophagy.

[32] Lamark T, Kirkin V, Dikic I, Johansen T (2009). NBR1 and p62 as cargo receptors for selective autophagy of ubiquitinated targets. Cell Cycle.

[33] Reggiori F, Klionsky DJ (2002). Autophagy in the eukaryotic cell. Eukaryot Cell.

[34] Schwartz-Roberts JL, Shajahan AN, Cook KL, Warri A, Abu-Asab M, Clarke R (2013). GX15-070 (obatoclax) induces apoptosis and inhibits cathepsin D- and L-mediated autophagosomal lysis in antiestrogen-resistant breast cancer cells. Mol Cancer Ther.

[35] Scherz-Shouval R, Shvets E, Fass E, Shorer H, Gil L, Elazar Z (2007). Reactive oxygen species are essential for autophagy and specifically regulate the activity of Atg4. EMBO J.

[36] Tripathi DN, Chowdhury R, Trudel LJ, Tee AR, Slack RS, Walker CL, Wogan GN (2013). Reactive nitrogen species regulate autophagy through ATM-AMPK-TSC2-mediated suppression of mTORC1. Proc Natl Acad Sci U S A.

[37] Clarke R, Cook KL, Hu R, Facey CO, Tavassoly I, Schwartz JL, Baumann WT, Tyson JJ, Xuan J, Wang Y, Warri A, Shajahan AN (2012). Endoplasmic reticulum stress, the unfolded protein response, autophagy, and the integrated regulation of breast cancer cell fate. Cancer Res.

[38] de la Cadena SG, Hernandez-Fonseca K, Camacho-Arroyo I, Massieu L (2013). Glucose deprivation induces reticulum stress by the PERK pathway and caspase-7- and calpain-mediated caspase-12 activation. Apoptosis.

[39] Haga N, Saito S, Tsukumo Y, Sakurai J, Furuno A, Tsuruo T, Tomida A (2010). Mitochondria regulate the unfolded protein response leading to cancer cell survival under glucose deprivation conditions. Cancer Sci.

[40] Davies MP, Barraclough DL, Stewart C, Joyce KA, Eccles RM, Barraclough R, Rudland PS, Sibson DR (2008). Expression and splicing of the unfolded protein response gene XBP-1 are significantly associated with clinical outcome of endocrine-treated breast cancer. Int J Cancer.

[41] Gomez BP, Riggins RB, Shajahan AN, Klimach U, Wang A, Crawford AC, Zhu Y, Zwart A, Wang M, Clarke R (2007). Human X-box binding protein-1 confers both estrogen independence and antiestrogen resistance in breast cancer cell lines. FASEB J.

[42] Bennett BL, Sasaki DT, Murray BW, O’Leary EC, Sakata ST, Xu W, Leisten JC, Motiwala A, Pierce S, Satoh Y, Bhagwat SS, Manning AM, Anderson DW (2001). SP600125, an anthrapyrazolone inhibitor of Jun N-terminal kinase. Proc Natl Acad Sci U S A.

[43] Chambliss KL, Yuhanna IS, Mineo C, Liu P, German Z, Sherman TS, Mendelsohn ME, Anderson RG, Shaul PW (2000). Estrogen receptor alpha and endothelial nitric oxide synthase are organized into a functional signaling module in caveolae. Circ Res.

[44] Chen X, Iliopoulos D, Zhang Q, Tang Q, Greenblatt MB, Hatziapostolou M, Lim E, Tam WL, Ni M, Chen Y, Mai J, Shen H, Hu DZ, Adoro S, Hu B, Song M, Tan C, Landis MD, Ferrari M, Shin SJ, Brown M, Chang JC, Liu XS, Glimcher LH (2014). XBP1 promotes triple-negative breast cancer by controlling the HIF1alpha pathway. Nature.

[45] Clarke R, Shajahan AN, Riggins RB, Cho Y, Crawford A, Xuan J, Zhang B, Facey C, Aiyer H, Cook K, Hickman FE, Tavassoly I, Verdugo A, Chen C, Zwart A, Wärri A, Hilakivi-Clarke LA (2009). Gene network signaling in hormone responsiveness modifies apoptosis and autophagy in breast cancer cells. J Steroid Biochem Mol Biol.

[46] Zhu Y, Singh B, Hewitt S, Liu A, Gomez B, Wang A, Clarke R (2006). Expression patterns among interferon regulatory factor-1, human X-box binding protein-1, nuclear factor kappa B, nucleophosmin, estrogen receptor-alpha and progesterone receptor proteins in breast cancer tissue microarrays. Int J Oncol.

[47] Benetatos L, Vartholomatos G, Hatzimichael E (2014). Polycomb group proteins and MYC: the cancer connection. Cell Mol Life Sci.

[48] Dang CV, Hamaker M, Sun P, Le A, Gao P (2011). Therapeutic targeting of cancer cell metabolism. J Mol Med (Berl).

[49] Zhao Y, Butler EB, Tan M (2013). Targeting cellular metabolism to improve cancer therapeutics. Cell Death Dis.

[50] Terunuma A, Putluri N, Mishra P, Mathe EA, Dorsey TH, Yi M, Wallace TA, Issaq HJ, Zhou M, Killian JK, Stevenson HS, Karoly ED, Chan K, Samanta S, Prieto D, Hsu TY, Kurley SJ, Putluri V, Sonavane R, Edelman DC, Wulff J, Starks AM, Yang Y, Kittles RA, Yfantis HG, Lee DH, Ioffe OB, Schiff R, Stephens RM, Meltzer PS (2014). MYC-driven accumulation of 2-hydroxyglutarate is associated with breast cancer prognosis. J Clin Invest.

[51] Dang CV (2013). MYC, metabolism, cell growth, and tumorigenesis. Cold Spring Harb Perspect Med.

[52] DeBerardinis RJ, Cheng T (2010). Q’s next: the diverse functions of glutamine in metabolism, cell biology and cancer. Oncogene.

[53] Wise DR, Thompson CB (2010). Glutamine addiction: a new therapeutic target in cancer. Trends Biochem Sci.

[54] Morrish F, Isern N, Sadilek M, Jeffrey M, Hockenbery DM (2009). c-Myc activates multiple metabolic networks to generate substrates for cell-cycle entry. Oncogene.

[55] Gaglio D, Soldati C, Vanoni M, Alberghina L, Chiaradonna F (2009). Glutamine deprivation induces abortive s-phase rescued by deoxyribonucleotides in k-ras transformed fibroblasts. PLoS One.

[56] Liu W, Le A, Hancock C, Lane AN, Dang CV, Fan TW, Phang JM (2012). Reprogramming of proline and glutamine metabolism contributes to the proliferative and metabolic responses regulated by oncogenic transcription factor c-MYC. Proc Natl Acad Sci U S A.

[57] Sehgal V, Ram PT (2013). Network motifs in JNK signaling. Genes Cancer.

[58] Noguchi K, Kitanaka C, Yamana H, Kokubu A, Mochizuki T, Kuchino Y (1999). Regulation of c-Myc through phosphorylation at Ser-62 and Ser-71 by c-Jun N-terminal kinase. J Biol Chem.

[59] Alarcon-Vargas D, Ronai Z (2004). c-Jun-NH2 kinase (JNK) contributes to the regulation of c-Myc protein stability. J Biol Chem.

[60] Toh PP, Luo S, Menzies FM, Rasko T, Wanker EE, Rubinsztein DC (2013). Myc inhibition impairs autophagosome formation. Hum Mol Genet.

[61] Vaupel P, Kallinowski F, Okunieff P (1989). Blood flow, oxygen and nutrient supply, and metabolic microenvironment of human tumors: a review. Cancer Res.

[62] Millon SR, Ostrander JH, Brown JQ, Raheja A, Seewaldt VL, Ramanujam N (2011). Uptake of 2-NBDG as a method to monitor therapy response in breast cancer cell lines. Breast Cancer Res Treat.

[63] Wirth M, Stojanovic N, Christian J, Paul MC, Stauber RH, Schmid RM, Häcker G, Krämer OH, Saur D, Schneider G (2014). MYC and EGR1 synergize to trigger tumor cell death by controlling NOXA and BIM transcription upon treatment with the proteasome inhibitor bortezomib. Nucleic Acids Res.

[64] Tisdale MJ (2009). Mechanisms of cancer cachexia. Physiol Rev.

[65] Holroyde CP, Skutches CL, Boden G, Reichard GA (1984). Glucose metabolism in cachectic patients with colorectal cancer. Cancer Res.

[66] Ko YH, Lin Z, Flomenberg N, Pestell RG, Howell A, Sotgia F, Lisanti MP, Martinez-Outschoorn UE (2011). Glutamine fuels a vicious cycle of autophagy in the tumor stroma and oxidative mitochondrial metabolism in epithelial cancer cells: implications for preventing chemotherapy resistance. Cancer Biol Ther.

